# Contrasting patterns of genetic and phenotypic differentiation in two invasive salmonids in the southern hemisphere

**DOI:** 10.1111/eva.12188

**Published:** 2014-07-23

**Authors:** Catalina Monzón-Argüello, Sofia Consuegra, Gonzalo Gajardo, Francisco Marco-Rius, Daniel M Fowler, Jacquelin DeFaveri, Carlos Garcia de Leaniz

**Affiliations:** 1 Department of Biosciences, Swansea UniversitySwansea, UK; 2 Laboratorio de Genética, Acuicultura y Biodiversidad, Universidad de Los LagosOsorno, Chile; 3 Ecological Genetics Research Unit, Department of Biosciences, University of HelsinkiHelsinki, Finland

**Keywords:** microsatellites, naturalization, *Oncorhynchus mykiss*, phenotypic divergence, *P*_ST_/*F*_ST_, rapid evolution, *Salmo trutta*, selection

## Abstract

Invasion success may be expected to increase with residence time (i.e., time since first introduction) and secondary releases (i.e., those that follow the original introduction), but this has rarely been tested in natural fish populations. We compared genetic and phenotypic divergence in rainbow trout and brown trout in Chile and the Falkland Islands to test the prediction that adaptive divergence, measured as *P*_ST_/*F*_ST_, would increase with residence time and secondary releases. We also explored whether interspecific competition between invaders could drive phenotypic divergence. Residence time had no significant effect on genetic diversity, phenotypic divergence, effective population size, or signatures of expansion of invasive trout. In contrast, secondary releases had a major effect on trout invasions, and rainbow trout populations mostly affected by aquaculture escapees showed significant divergence from less affected populations. Coexistence with brown trout had a positive effect on phenotypic divergence of rainbow trout. Our results highlight an important role of secondary releases in shaping fish invasions, but do not support the contention that older invaders are more differentiated than younger ones. They also suggest that exotic trout may not have yet developed local adaptations in these recently invaded habitats, at least with respect to growth-related traits.

## Introduction

Understanding the tempo and mode of biological invasions is important for minimizing the potential impacts of invasive species (Kolar and Lodge 2001, 2002; Marchetti et al. 2004). Theory predicts that invasion success will often depend on three main factors: (i) *propagule pressure*, that is, the number of dispersing individuals and the number of discrete release events, as these determine standing genetic variation and provide adaptive potential (Barrett and Schluter 2008); (ii) *species invasiveness*, that is, those traits that enable a species to invade novel habitats; and (iii) *invasibility* of the recipient community, that is, the susceptibility of communities to be invaded (Lonsdale 1999; Alpert et al. 2000; Lockwood et al. 2005). Consensus is also growing on the importance of *residence time* (i.e., time since a population became established) and *secondary releases* (i.e., those that follow the original introduction, usually at different locations) in determining invasion success. Among invasive plants, residence time and secondary releases often promote naturalization and population expansion (Kowarik 2003; Wilson et al. 2007; Dlugosch and Parker 2008; Dainese and Poldini 2012), yet their role on animal invasions remains largely unexplored.

Residence time represents another dimension of propagule pressure; in general, the longer the time has passed since the initial introduction, the more propagules will spread, thereby increasing the probability of founding new populations (Pyšek and Jarošík 2005). Secondary releases, on the other hand, often facilitate invasions by increasing the genetic variation in introduced populations (Kowarik 2003; Perrings et al. 2005), which would otherwise be expected to exhibit reduced genetic diversity as a consequence of a typically low number of founders (Nei et al. 1975). The effect of secondary releases on invasion success is particularly strong when these involve different source populations released at different geographical locations (Ellstrand and Schierenbeck 2000; Bossdorf et al. 2005) as these will have more chances of becoming established (Novak and Mack 2005; Crawford and Whitney 2010), and subsequent admixture and hybridization among multiple independent introductions may increase the level of standing genetic variation (Lee 2002; Kolbe et al. 2004; Lavergne and Molofsky 2007; Prentis et al. 2008), extending the window of opportunity for invasions to occur (Dlugosch and Parker 2008).

Introduced species are expected to be poorly adapted to novel environments and will likely encounter novel selection pressures during invasions (Facon et al. 2006). While phenotypic plasticity may facilitate initial establishment (Richards et al. 2006), the effects of selection might be expected to become more important during the subsequent invasion stages (Keller and Taylor 2008), when selection can drive the evolution of phenotypic plasticity (Lande 2009) and generate potential for rapid evolution – that is changes in adaptive traits occurring within 20 or fewer generations (Thompson 1998; Reznick and Ghalambor 2001; Prentis et al. 2008). Hence, phenotypic changes are a likely outcome of the invasion process (Bossdorf et al. 2005) with phenotypic divergence between ancestral and invasive lineages thought to be determined by prior evolutionary history, chance events, and response to selection (Keller and Taylor 2008). Measuring how invasive species respond to new selection pressures remains challenging, and examining adaptive divergence might be a step forward. Adaptive divergence can be inferred by comparing phenotypic (*P*_ST_; Spitze 1993) and neutral genotypic (*F*_ST_) differentiation (Merilä and Crnokrak 2001). In theory, when *P*_ST_ ≠ *F*_ST_, drift alone would be insufficient to explain observed phenotypic divergence, and divergent (*P*_ST_ > *F*_ST_) or convergent (*P*_ST_ < *F*_ST_) selection on the trait of interest may be invoked (Leinonen et al. 2008). However, one of the potential limitations of using *P*_ST_ to estimate additive genetic variance is that it can be confounded by environmental and nonadditive genetic effects (Pujol et al. 2008; Brommer 2011), so caution is needed on its interpretation.

**Figure 1:**
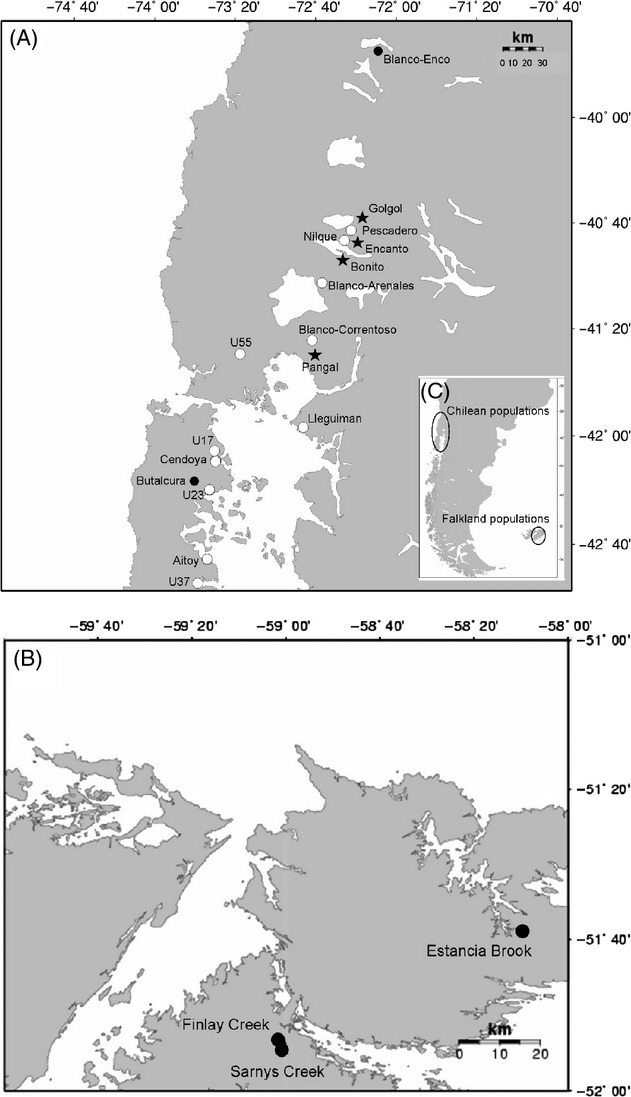
Study populations of brown trout (closed circles) and rainbow trout (open circles) in (A) Chile and (B) the Falkland Islands. Stars represent rivers sampled for both species.

We compared patterns of invasion and divergence of two exotic salmonids, rainbow trout (*Oncorhynchus mykiss*) and brown trout (*Salmo trutta*), in two locations in the southern hemisphere, Chilean Patagonia and the Falkland Islands (Fig. 1). The two species rank among the most successful aquatic invaders in the world (Lowe et al. 2000; Garcia de Leaniz et al. 2010) and occupy similar niches in different continents (rainbow trout in the Pacific coast of North America, brown trout in Europe). They have now converged in novel geographical ranges in South America (Crawford and Muir 2008; Garcia de Leaniz et al. 2010; Young et al. 2010), where they tend to dominate the fish communities of numerous streams and lakes across Patagonia, having become fully naturalized (i.e., self-sustained) in most cases (Young et al. 2010; Habit et al. 2012). Of the two species, brown trout tends to display lower invasiveness (i.e., narrower geographic range) but a stronger impact on native fishes (Young et al. 2010; Correa and Hendry 2012). In addition, brown trout has been dispersed mostly through stocking and natural colonization, whereas the spread of rainbow trout has been facilitated by the escape of farmed fish since the 1990s following the rapid expansion of the Chilean salmon industry (Gajardo and Laikre 2003; Arismendi et al. 2009). Rainbow trout originating from such secondary releases survives and interbreeds with naturalized populations, and this may have helped to spread the species across much of Patagonia (Consuegra et al. 2011).

We used data on phenotypic and molecular variation in two salmonid invaders to test two predictions, namely that (i) older populations with longer residence time would display stronger genetic and phenotypic differentiation than younger, more recent populations of each species and (ii) that populations aided by secondary releases would be more differentiated than those that have dispersed mostly through natural colonization.

## Material and methods

### Origin of study populations

Brown trout is native to Eurasia and rainbow trout to the West coast of North America, but both species were reared in hatcheries and propagated for sport fishing and aquaculture to many countries elsewhere (MacCrimmon and Marshall 1968; Crawford and Muir 2008). Rainbow trout and brown trout were first introduced successfully into Chile in 1905, probably from Hamburg in Germany (MacCrimmon and Marshall 1968; MacCrimmon 1971; Garcia de Leaniz et al. 2010). The success of earlier introductions (1883–1888) cannot be ascertained (Basulto 2003). Rainbow trout had been imported from the USA to German hatcheries on several different occasions during 1882–1928 (MacCrimmon 1971) and these included migratory steelhead from California, which may have been the strain later shipped to Chile. From Chile, both species were then shipped to the Falkland Islands during 1936–1947, but only brown trout survived (Arrowsmith and Pentelow 1965).

In contrast to brown trout, which in Chile seems to be dominated by strains of German origin (MacCrimmon and Marshall 1968; Faundez et al. 1997), rainbow trout has a much more varied origin (Colihueque et al. 2001), as in addition to the original US shipments via Germany, at least 17 additional commercial strains from four different countries have been introduced in more recent times (Table S1): Denmark (57% of imported eggs), USA (25% of eggs), Norway (17% of imported eggs), and Finland (1% of imported eggs). These include freshwater strains (e.g., Troutex Trachsel, AquaSearch Fresh, AquaSearch Late, Troutlodge Kamloops) as well as strains selected for high salinity tolerance adapted for life in seawater (e.g., AquaSearch Salt, Troutlodge Silver Steelhead, SalmoBreed). Although we were not able to identify the original North American locations of these European rainbow trout stocks, they likely came from different sources, making the origin of rainbow trout in Chile potentially more varied than brown trout.

Brown trout introduced in the Falklands is thought to have originated from the same two broad origins as in Chile, from a German origin shipped via Chile and the United States (McDowall et al. 2001) and from a British origin, including English (Surrey, Lancashire) and Scottish sources (Pentlands, Table S1). Until 2013, when c. 10 000 sea trout smolts derived from local broodstock were transferred to sea cages at Fitzroy Sound, there had not been any trout farming or intentional releases of trout in the Falkland Islands; hence, all brown trouts are thought to have been the result of natural colonization following the initial 1936–1962 stocking (Stewart 1973). As with rainbow trout, brown trout stocked into Chile and the Falkland Islands includes the progeny of both resident and anadromous (sea trout) parents. Thus, rainbow trout in Chile has been affected by secondary releases much more than brown trout, and brown trout in Chile has had much longer residence time than in the Falkland Islands, despite sharing the same two broad origins (Germany and Britain).

### Sampling

We analyzed 314 wild (i.e., free-living) rainbow trout collected from 15 streams in Chile and 187 wild brown trout collected from six stream Chile and three streams in the Falkland Islands during 2007–2009 (Fig. 1). Details of the first to third-order study streams are given in Vanhaecke et al. (2012a) for Chile and Vanhaecke et al. (2012b) for the Falkland Islands. Fish were collected by a combination of single-pass electrofishing (LR-24; Smith-Root Corporation, Vancouver, WA, USA) and angling (one stream) close to the river mouths, as these represent the main invasion routes for exotic trout in the area (Consuegra et al. 2011). Scale samples, fork length (measured from the tip of the snout to the fork of the tail; FL, mm), and wet weight (W, g) were available for a subsample of 136 rainbow trout and 107 brown trout from seven and five populations, respectively (Table 1). Fish age was determined by counting the number of annuli.

**Table 1:** Genetic diversity of brown trout (*n* = 9 populations) and rainbow trout (*n* = 15 populations) in Chilean Patagonia and the Falkland Islands

Species	Population	*N*_*g*_	*N*_*p*_	*N*_*A*_	AR_10_/AR_9_	*H*_*o*_	*H*_*e*_	*F*_IS_	*N*_*e*_ (95CI)	*J'*	Age
Brown trout											
Chile	Golgol*	21	–	5.57	4.80/4.67	0.66	0.66	−0.005	38 (22,75)	–	Old
	Butalcura	22	22	4.64	4.19/4.11	0.61	0.63	0.027	36 (21,73)	–	Old
	Blanco-Enco	19	19	5.07	4.46/4.35	0.66	0.65	−0.015	23 (13,47)	–	New
	Pangal*	23	23	4.14	3.78/3.72	0.62	0.62	−0.006	34 (20,66)	–	Old
	Encanto*	21	21	5.21	4.56/4.44	0.62	0.65	0.050	35 (19,81)	–	Old
	Bonito*	20	–	5.21	4.69/4.59	0.68	0.67	−0.015	32 (18,64)	–	New
Falklands	Estancia Brook	23	11	7.93	6.44/6.21	0.72	0.76	0.055	46 (26,91)	–	Old
	Finlay Creek	23	–	2.79	2.57/2.54	0.41	0.41	0.007	17 (9,38)	–	New
	Sarnys Creek	15	–	3.14	2.91/2.84	0.41	0.41	−0.001	16 (8,39)	–	New
Rainbow trout											
Chile	*Blanco-Correntoso*	20	20	6.86	NA/5.64	0.63	0.73	0.144	20 (10,48)	0.67	Old
	**Pescadero**	30	23	8.57	NA/5.96	0.66	0.76	0.132	23 (13,48)	0.82	Old
	*Nilque*	30	22	7.14	NA/5.56	0.63	0.74	0.163	25 (13,52)	0.22	Old
	Pangal*	18	16	7.00	NA/5.78	0.73	0.74	0.008	35 (16,133)	0.99	New
	Encanto*	26	23	8.29	NA/6.12	0.70	0.76	0.106	20 (11,41)	0.47	Old
	Lleguiman	14	–	7.71	NA|/6.59	0.82	0.78	−0.021	25 (13,54)	0.76	New
	Blanco-Arenales	15	–	6.71	NA/5.65	0.63	0.71	0.128	22 (10,68)	0.57	New
	U17	18	–	6.43	NA/5.08	0.68	0.69	0.047	29 (15,74)	0.76	Old
	**U23**	17	17	7.29	NA/6.37	0.74	0.77	0.073	29 (15,88)	0.98	New
	U37	13	–	5.00	NA/4.57	0.58	0.63	0.092	25 (10,45)	0.76	New
	**Aitoy**	16	16	7.71	NA/6.79	0.77	0.80	0.068	52 (20,130)	0.88	New
	U55	9	–	5.00	NA/5.00	0.78	0.72	−0.028	23 (10,182)	0.62	New
	Golgol*	29	–	7.29	NA/5.29	0.70	0.72	0.038	22 (12,42)	0.21	Old
	Bonito*	29	–	8.43	NA/6.17	0.68	0.77	0.140	22 (12,43)	0.36	Old
	Cendoya	30	–	4.86	NA/3.79	0.55	0.57	0.041	16 (8,34)	0.00	Old

Estimates of effective population size (*N*_e_) and their 95% confidence intervals using the full likelihood method implemented in colony (Jones and Wang 2010) are included, as well as inferred age of the populations. Pielou's evenness index (*J'*) represents the extent of admixture of individuals belonging to each genetic cluster as detected by structure. Rainbow trout populations with high (*J*' = 0.82–0.99) or moderate (*J*' = 0.22–0.67) levels of admixture with farmed fish are denoted in bold or italics, respectively.

*N*_*g*_, sample size for genetic analysis; *N*_*p*_, sample size for phenotypic analysis *N*_*A*_, number of observed alleles; AR_10_ allelic richness based on 10 diploid individuals for comparisons among brown trout populations; AR_9_ allelic richness based on nine diploid individuals for comparisons between rainbow trout and brown trout; *H*_*o*_, observed heterozygosity; *H*_*e*_, expected heterozygosity; *F*_IS_, inbreeding coefficient.

Streams where brown trout and rainbow trout coexist are denoted with an asterisk (*).

### DNA extraction and microsatellite amplification

#### Rainbow trout

All rainbow trout had previously been genotyped for seven microsatellite loci, and the extent of admixture with secondary releases from farmed escapees had been estimated for each study river (details in Consuegra et al. 2011).

#### Brown trout

Total genomic DNA was extracted from brown trout samples with the Wizard® SV96 Genomic DNA purification kit (Promega, Madison, WI, USA) following the manufacturer's instructions and 14 di- and tetranucleotide microsatellites were PCR amplified (details deposited in Figshare doi:10.6084/m9.figshare.953191). These included 12 putatively neutral markers – *Str15, Str60*, *Str73* (Estoup et al. 1993)*, Ssa408, Ssa410UoS* (Cairney et al. 2000), *BG935488* (Vasemagi et al. 2005), *SsoSL417* (Slettan et al. 1995), *SsaF43* (Olafsson et al. 2010), *SsaD71* (King et al. 2005), *Ssa171, Ssa197* (O'Reilly et al. 1996), *ppStr3* (Prodöhl pers. comm.) – and two markers (*MHCI and TAP2A*) tightly linked to the MHC class I and TAP genes, respectively (Grimholt et al. 1993, 2002). As there were no differences in summary statistics whether the markers were analyzed together (*n* = 14) or separately (neutral: *n* = 12; gene-linked: *n* = 2; Table S3a,b), all analyses were performed with the total set of 14 markers.

PCR amplifications were carried out in two multiplex reactions (eight and six microsatellites, respectively) of 11 μL, using the QIAGEN Multiplex PCR kit (Qiagen, Sussex, UK) and 3 μL of extracted DNA (∼20 ng), following the manufacturer's recommendations. PCR products were diluted 1:10 in water and resolved on an Applied Biosystems ABI3130xl Genetic Analyser (Applied Biosystems, Sussex, UK). Fragment length was determined using the GeneScan 500-LIZ size standard and scored using genemapper 4.0 (Applied Biosystems, Paisley, UK).

### Genetic diversity

Allelic data for brown trout were screened for genotyping errors using micro-checker 2.2.3 (Van Oosterhout et al. 2004) and full genotypes deposited in figshare (doi: 10.6084/m9.figshare.953191). Deviations from Hardy–Weinberg equilibrium (HWE), for each study site and locus, as well as linkage disequilibrium for each pair of loci, were estimated using genepop 3.4, and significance values were adjusted by a sequential Bonferroni correction. As two of the microsatellites were linked to the *MHCI* and *TAP2A* genes, respectively (both related to the immune response, Grimholt et al. 1993, 2002), we investigated signatures of selection using lositan (Antao et al. 2008). For all runs, 10 000 simulations were generated both under the infinite alleles and stepwise mutation model with ‘neutral mean *F*_ST_’ and ‘forced mean *F*_ST_’. We also used bayescan 2.0 (Foll and Gaggiotti 2008) to estimate the posterior probability that each locus was subject to selection. Population genetic diversity was evaluated by number of alleles (*N*_*A*_), allelic richness based on 10 diploid individuals (AR_10_; fstat 2.9.3; Goudet 1995), observed heterozygosity (*H*_*o*_), and unbiased expected heterozygosity (*H*_*e*_; Genetix 4.0; Belkhir et al. 2001 Nei 1987). Differences in diversity, relatedness, and *F*_IS_ values between locations were assessed in fstat using 1000 permutations. For comparisons with rainbow trout, AR was recalculated based on nine diploid individuals (AR_9_).

Estimates of effective population sizes (*N*_e_) of brown trout were obtained by two different methods, using a full likelihood method based on sibship assignments and random mating implemented in colony 2.0 (Jones and Wang 2010) and using an approximate Bayesian computation implemented in onesamp (Tallmon et al. 2008). To investigate potential demographic changes associated with variation in residence time and secondary releases, we examined evidence of genetic signatures of population contraction (bottlenecks) or expansion. Evidence for recent population bottlenecks was assessed by one-tailed Wilcoxon tests of heterozygosity excess in Bottleneck 1.2 (Cornuet and Luikart 1996), using 10 000 iterations and a two-phase model of mutation (TPM). Evidence for population expansion was assessed by examining deviations from the mutation-drift equilibrium using the intralocus *k*-test and the interlocus *g*-test (Reich et al. 1999) in kgtests (Bilgin 2007). The statistical significance of the *g* value in the kgtests was assessed at *α* = 0.05 for a given number of loci and sample sizes according to Reich et al. (1999).

### Genetic differentiation

Genetic differentiation between samples was calculated for each species using pairwise *F*_ST_ in fstat and the unbiased estimator *D*_est_ (Jost 2008) in smogd 1.2.5 (Crawford 2010). Significance was assessed with 10 000 permutations. We tested for isolation by distance (IBD) using a Mantel test implemented in arlequin 3.5 (Excoffier and Lischer 2010) and 100 000 permutations. To further investigate population structure, we used the model-based clustering method implemented in structure 2.3.3 (Pritchard et al. 2000). For each *K* (ranging from *K* = 2 to *K* = 10), we computed 100 iterations with a burn-in of 25 000 and 75 000 MCMC replicates using the admixture model with allele frequencies correlated. To assess the most likely number of clusters, we calculated Δ*K* following Evanno et al. (2005). We also used tess 2.3 (Chen et al. 2007), which includes spatial information, to determine the most likely number of cluster considering the deviance information criterion (lowest DIC value; Spiegelhalter et al. 2002).

A hierarchical analysis of molecular variation (amova) was used to partition variation among- and within-population components using the program arlequin v3.5 (Excoffier and Lischer 2010). Hierarchies considered for brown trout were (i) broad geographical location (i.e., Chile versus Falkland Islands), (ii) clusters identified by structure and tess, and (iii) relative residence time (i.e., population age) inferred from the presence/absence of native galaxiid fishes. Given that brown trout tends to drive native galaxiids to extinction (reviewed in McDowall 2006), we assumed that the absence of the native galaxiids *Aplochiton* sp. and *Galaxias* sp. would be associated with older trout invasions. Very little is known about the extinction process of galaxiids invaded by salmonids, as accurate information on the date and precise locations of first introduction is rare; however, studies indicate that local extirpations following salmonid invasions can be rapid. For example, in the Falkland Islands, *Aplochiton* sp. have become extinct in some rivers within 50–60 years of the first introduction of brown trout (McDowall et al. 2001; McDowall 2006), and we used this figure as a rough cutoff point to classify brown trout populations as ‘old’ (>60 years) or ‘new’ (<60 years).

In the case of rainbow trout, hierarchies considered in the amova included (i) level of admixture with farm fish (moderate versus high according to Pielou's *J'* evenness index – Consuegra et al. (2011): high, *n* = 4; *J*' = 0.82–0.99; moderate, *n* = 3; *J*' = 0.22–0.67; Table 1), (ii) clusters identified by structure and tess, (iii) coexistence with brown trout (present versus absent), and (iv) relative residence time (age of each population). Residence time was inferred from the relative abundance of ‘aquaculture alleles’ in the population using a median cutoff point of 0.6 to classify populations as ‘recent’ (*q* > 0.6) or ‘old’ (*q* ≤ 0.6; see Consuegra et al. 2011). We assumed that recent rainbow populations would show more introgression from aquaculture escapees than older ones, given that large-scale farming of rainbow trout is a relative recent activity in Chile (Gajardo and Laikre 2003) and that the genetic diversity of trout escapees tends to decrease with time spent in the wild (Monzón-Argüello et al. 2013).

### Phenotypic differentiation

We estimated phenotypic differentiation (*P*_ST_) between seven populations of rainbow trout and five populations of brown trout at four size-related phenotypic traits that are likely to show divergence during the colonization of novel geographical ranges with different growing conditions: (i) condition factor (Blackwell et al. 2000), (ii) number of scale growth circuli during the first winter, (iii) scale intercirculi spacing during the first winter, and (iv) scale radius at the end of the first winter, as detailed in Marco-Rius et al. (2012, 2013). Analysis of scale growth circuli can be used to reconstruct and compare growth profiles of individuals of different ages and has previously been used to assess variation in growth performance of invasive trout in the area (Schröder and Garcia de Leaniz 2011). Size- and growth-related traits tend to be heritable in salmonids (mean *h*^2^ = 0.25 for size and growth rate, mean *h*^2^ = 0.23 for condition factor; Garcia de Leaniz et al. 2007; Carlson and Seamons 2008) and are thus likely to respond quickly to novel selection pressures during fish invasions.

Repeatability in scale measurements was assessed by comparing the measurements of the first winter scale radius of 30 trouts of each species measured by two observers working independently after discarding the first two circuli to minimize bias due to scale erosion (Marco-Rius et al. 2012). Repeatability in scale length at the end of the first year was calculated as the agreement intraclass correlation coefficient (ICC) with the ‘psy’ R package, defined as the ratio of the subject variance divided by the sum of the subject variance, the observer variance, and the residual variance (Wolak et al. 2012). Repeatability for this trait was high for both species (brown trout = 0.77; rainbow trout = 0.92). Repeatabilities for the other scale traits were not calculated as we had previously found that these were similarly high in brown trout (Marco-Rius et al. 2012, 2013).

### Statistical analysis

Differences in genetic diversity and phenotypic traits among populations were calculated for each species using one-way anova. We employed REML linear mixed-effects model implemented in ‘nlme 3.1-86’ package (Pinheiro and Bates 2009) in r 2.14 language (R Development Core Team, 2008) and the Akaike information criteria (AIC) to model variation in intercirculi spacing using population of origin, fork length, and age as fixed factors, and fish ID and circulus number as random factors, as described in Marco-Rius et al. (2013). These were assumed to be independent among individuals, and to follow a normal distribution with mean zero and variances 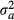 and 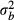, respectively, the observation error *ε*_*i,j*_ was also assumed to be independent and normally distributed. We tested for random effects in the model and allowed for autocorrelation in intercirculi spacing by considering an autoregressive term of order one, as this provided a better fit than a model without autocorrelation.

Divergence was inferred by comparing phenotypic differentiation (*P*_ST_) with neutral molecular differentiation (*F*_ST_; Merilä and Crnokrak 2001) following the method of Whitlock and Guillaume (2009), which uses the distribution of differentiation at neutral loci to simulate the distribution of *P*_ST_ expected under neutrality. Random values of *F*_ST_, *h*^2^, 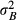, and 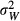 were used to simulate the null distribution of *P*_ST_/*F*_ST_ (obtained from 10 000 iterations; see below for details on how values were obtained). The observed value of *P*_ST_/*F*_ST_ was then compared with the simulated *P*_ST_/*F*_ST_ to determine whether it fell outside the simulated distribution. Computations were performed in R following the codes provided by Holand et al. (2011), which incorporate additive genetic variance within (*h*^2^) and between (*c*) populations (Brommer 2011). Briefly, as the traits under investigation were measured from wild-caught individuals, it is impossible to determine how much of the observed phenotypic variation is due to environmental or genetic effects (Brommer 2011). Therefore, a scalar (*c*) was included to allow for environmental between-population variance, whereby small values of *c* (e.g., close to 0) indicate that the phenotypic variation is mostly influenced by environmental effects, and large values of *c* (e.g., close to 1) indicate that only genetic variation has contributed to phenotypic differences. We estimated *P*_ST_ according to Brommer (2011), as 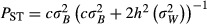, where *h*^2^ represents the trait-specific heritability, *c* represents the additive gene proportion among populations, and 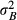 and 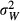 represent the among- and within-population variance, respectively.

Simulated values of *F*_ST_ were obtained by randomly sampling from the bootstrap distribution of mean *F*_ST_, generated from bootstrapping loci 10 000 times with the r package hierfstat (Goudet 2005). Simulated values of among- and within-population variations were obtained by multiplying 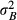 (*a*–1)^−1^ with a random number drawn from the chi distribution having (*a*–1) degrees of freedom (*a* being the number of populations). Samples of *F*_ST_, *h*^2^, 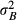
_,_ and 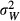 were randomly drawn and used to calculate a simulated *P*_ST_/*F*_ST_. This was repeated 1000 times to generate a sampling distribution of *P*_ST_/*F*_ST_ under neutrality, which was then used to compare observed *P*_ST_/*F*_ST_ for different values of *c,* ranging from 0 to 1. The critical value for *c* (where observed *P*_ST_/*F*_ST_ was larger than 95% confidence interval) was determined, and if this was less than *h*^2^, *P*_ST_ was deemed to be significantly higher than expected under neutrality (Brommer 2011). The critical value of *c* was then used in the *P*_ST_ equation given above to calculate pairwise *P*_ST_ values, which were then used to explore the effects of admixture, residence time, and coexistence with brown trout on phenotypic divergence. They were also used to test whether *P*_ST_ or *P*_ST_/*F*_ST_ were associated with geographical distance. For brown trout, *P*_ST_/*F*_ST_ was calculated among Chilean populations (*n* = 4) as well as between Chilean and Falkland populations (*n* = 5). For rainbow trout (which is absent in the Falklands), *P*_ST_/*F*_ST_ was calculated among all populations (*n* = 7; Table 1).

## Results

### Brown trout

#### Quality of genetic data

Three microsatellite loci (*Str73*, *SsoSL417* and *SsaD71*) had some missing data in the Falklands populations and one tetra-nucleotide marker (*Ssa171)* presented some alleles that differed only in two base pairs, probably due to the compound motif GTGA + GT. The exclusion of these markers did not change our results; hence, we retained them in the analyses. No evidence of null alleles, stuttering, or allele dropout was detected for any microsatellite, and no marker deviated significantly from HWE after sequential Bonferroni correction in more than one population (data not shown). Analysis of linkage disequilibrium was significant in only three of 819 pairwise comparisons (between loci and populations). There was no evidence for outliers under divergent selection with either lositan or bayescan (Table S3a).

#### Genetic diversity

**Figure 2:**
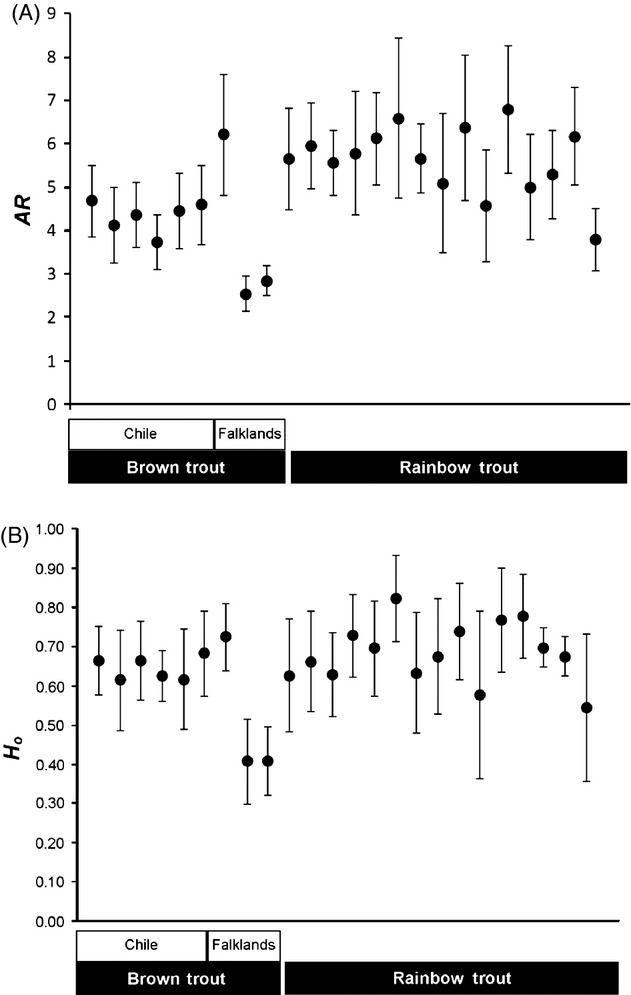
Microsatellite genetic diversity measure as (A) allelic richness based on nine diploid individuals (AR_9_) and (B) observed heterozygosity (*H*_*o*_) in brown trout and rainbow trout populations. Bars represent 95% confidence intervals and populations are represented, from left to right, in the same order as in Table 1.

Diversity estimates (NA, AR, *H*_*o*_, *H*_*e*_) were varied among populations, with a tendency to be lower in the Falkland Islands than in Chile (with the exception of Estancia Brook; Table 1). Allelic richness (AR_10_) and observed heterozygosity (*H*_*o*_) were significantly different between all brown trout populations (AR_10_: anova *F*_8,117_ = 6.517, *P* < 0.001; *H*_*o*_: anova *F*_8,117_ = 4.848, *P* < 0.001; Table 1, Fig. 2), but these were unrelated to broad geographical location, and no differences were found between Chile (mean AR_10_ = 4.41; mean *H*_*o*_ = 0.644) and the Falkland Islands (mean AR_10_ = 3.97; mean *H*_*o*_ = 0.526) considered as a whole (*P* = 0.660 and *P* = 0.120, respectively). Similarly, we found no significant differences in relatedness (Chile *r* = 0.159; Falklands *r* = 0.373; *P* = 0.302), *F*_IS_ (Chile *F*_IS_ = 0.006; Falklands *F*_IS_ = 0.034; *P* = 0.243) or global *F*_ST_ among populations (Chile *F*_ST_ = 0.087; Falklands *F*_ST_ = 0.235; *P* = 0.289), although *D*_est_ values were lower in Chile (*D*_est_ = 0.237, 95% CI = 0.169–0.310) than in the Falklands (*D*_est_ = 0.355, 95% CI = 0.239–0.472; Table S4). However, it should be noted that the low number of populations analyzed in the Falklands, combined with their different origins (see Results from structure below), could limit our ability to detect significant differentiation between Chile and the Falklands.

Our two estimates of effective population size (*N*_e_) using colony and onesamp were highly correlated (*r* = 0.82, *P* = 0.007) and yielded small sizes (*N*_e_ < 50) for all brown trout populations in all cases (Table 1). The program Bottleneck showed a heterozygosity excess in one of the Chilean populations (R. Bonito, *P* = 0.025), characterized by negative *F*_IS_ values (*F*_IS_ = −0.015), while population expansion was only detected in one Falkland population (Sarnys Creek, *P* = 0.022).

**Figure 3:**
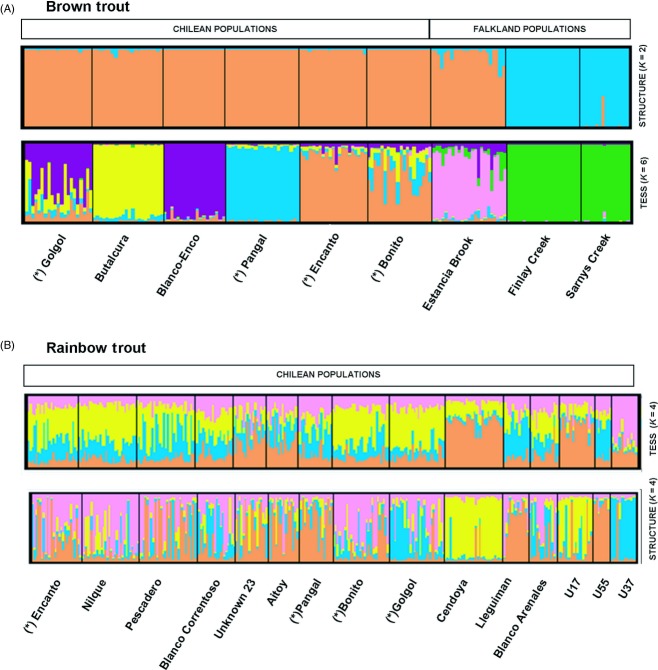
Bayesian clustering analyses of (A) brown trout and (B) rainbow trout populations according to structure and tess assuming two and six inferred clusters for brown trout (*K* = 2 and *K* = 6) and four inferred cluster for rainbow trout (*K* = 4). Each vertical bar represents an individual, with colours representing the probability of membership to each of the clusters. Asterisks show rivers sampled for both species.

**Figure 4:**
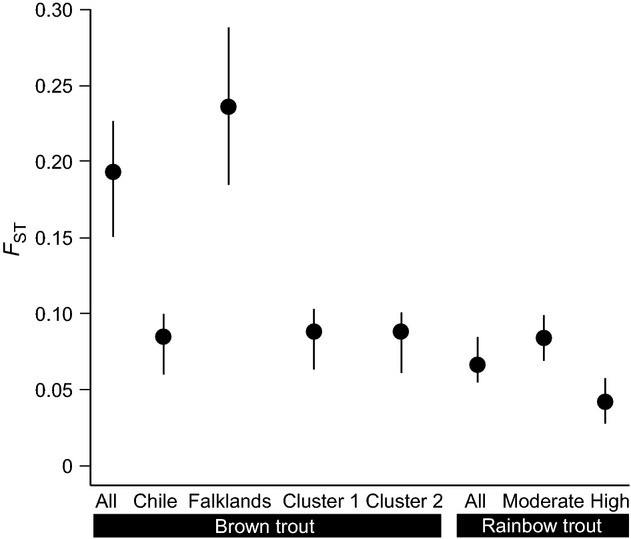
Level of genetic differentiation (*F*_ST_) among populations classified according to geographical location or assignment to genetic cluster (brown trout), and level of admixture (moderate or high) in rainbow trout. Bars represent 95% confidence intervals.

Pairwise *F*_ST_ values ranged from 0.044 (among Chilean populations) to 0.390 (between Chilean and Falkland Island populations; Table S4a). Pairwise *D*_est_ values were positively correlated with *F*_ST_ values (*r* = 0.391, *P* = 0.02; Table S4a). Pairwise *F*_ST_ values estimated with neutral and gene-linked markers were strongly correlated with pairwise differentiation estimated with all markers combined (*r* = 0.997, *P* < 0.001; *r* = 0.659, *P* < 0.001, respectively; Table S4b). There was no evidence of IBD (*z* = 162130.02, *r* = 0.090, *P* = 0.398). structure showed two genetic clusters (*K* = 2), but these did not exactly match with the two broad geographical areas analyzed. The first cluster included all Chilean populations and Estancia Brook (in the Falklands), while the second cluster comprised the two other Falkland populations (Fig. 3). Differentiation within each cluster was similar (Fig. 4). These results were supported by amova, which revealed a significant proportion of variation among groups (27.09%; Table 2). Results from tess suggested a finer pattern of structuring (*K* = 6), splitting the first cluster identified by structure into five independent clusters (Fig. 3). Individual assignments based on *K* = 6 indicated that, in general, each population had a very uniform genetic background except for two Chilean populations (R. Gol-Gol and R. Bonito; Fig. 3), which showed evidence of admixture. This was also supported by amova, which revealed a significant percentage of variation among groups (16.96%; Table 2), which was smaller than variation observed among structure clusters. Relative age of the populations, a proxy for residence time inferred by the presence or absence of native galaxiid fishes, did not explain a significant amount of molecular variation (3.26%; Table 2).

**Table 2:** Amount of molecular variation (%) among groups of brown trout and rainbow trout according to various hierarchies (Vg, among groups; Vp, among populations within groups; Vw, within populations). Figures in bold account for statistically significant variation (*P* < 0.05)

	Molecular variation (%)		
	
Species/Hierarchical comparison	Vg	Vp	Vw
Brown trout			
Location (Chile versus Falkland Islands)	**14.83**	**11.5**	**73.68**
structure cluster *K* = 2	**27.09**	**6.98**	**65.93**
tess cluster *K* = 6	**16.96**	**4.42**	**78.62**
Residence time (age of population) Old versus new	3.26	**18.29**	**78.46**
Rainbow trout			
Secondary releases Moderate versus high admixture	0.02	**7.29**	**92.69**
structure/tess cluster *K* = 4	**2.72**	**5.22**	**92.06**
Residence time (age of population) Old versus new	0.79	**6.90**	**92.31**
Coexistence with brown trout (BT) BT present versus absent	**1.76**	**6.45**	**91.79**

#### Phenotypic differentiation

Brown trout ranged between 47 and 556 mm in fork length; the minimal adequate mixed-effects model that explained variation in the spacing between consecutive scale growth rings included population as the only significant fixed term (*F*_4,86_ = 6.90, *P* < 0.001; Figure S1A). We also found significant differentiation among populations with respect to condition factor (*F*_3, 78_ = 34.34, *P* < 0.001), number of circuli deposited in the scales during the first freshwater year (*F*_4, 90_ = 6.78, *P* < 0.001), and scale size at the end of first year (*F*_4, 90_ = 5.41, *P* < 0.001).

#### *P*_ST_/*F*_ST_ comparisons

The contribution of environmental effects to phenotypic variance was low only for condition factor, as evidenced by the fact that *P*_ST_/*F*_ST_ was significantly higher than the neutral expectation for most values of *c* for this trait (Table 3; Figure S2A). However, while the inferences of our *P*_ST_ estimates are likely to be robust because *c* < *h*^2^ for this trait (Brommer 2011; Mobley et al. 2011), divergence in condition factor does not appear to be driven by residence time as it was significant when analyzed without the Falkland population. For the other traits, whether analyzed with all populations (e.g., between Chilean and Falkland populations) or only among Chilean populations, the observed *P*_ST_/*F*_ST_ was not significantly different from the simulated *P*_ST_/*F*_ST_ for most values of *c* (Table 3), likely indicating a strong environmental component to the observed patterns. *P*_ST_ for the four phenotypic traits was not correlated with either neutral *F*_ST_ or geographical distance (Table S5). Similarly, variation in *P*_ST_/*F*_ST_ was unrelated to geographical distance for all traits (Table S5).

**Table 3:** Critical *c* values for which the observed *P*_ST_/*F*_ST_ values are smaller (*c* ≤ 0.025) or larger (*c* ≥ 0.975) than expected under neutrality for four size-related phenotypic traits in brown trout and rainbow trout (condition factor; scale intercirculi spacing during the first winter; scale radius at the end of the first winter; number of scale growth circuli during the first winter). Figures in bold indicate those for which *c* < *h*^2^

Species/Trait comparison	Lower than expected (*c* ≤ 0.025 quantile)	Higher than expected (*c* ≥ 0.975 quantile)
Brown trout – Chile and Falklands		
Condition factor	NA	NA
Inter-circuli spacing at first winter	0.087	0.999
Scale radius at the end of first winter	0.015	0.321
No. of growth circuli during first winter	0.023	0.489
Brown trout – Chile		
**Condition factor**	0.002	**0.084**
Inter-circuli spacing during first winter	0.041	0.999
Scale radius at the end of first winter	0.015	0.461
No. of growth circuli during first winter	0.011	0.517
Rainbow trout – Chile		
**Condition factor**	0.002	**0.033**
**Inter-circuli spacing during first winter**	0.003	**0.047**
**Scale radius at the end of first winter**	0.018	**0.230**
**No. of growth circuli during first winter**	0.011	**0.143**

### Rainbow trout

#### Genetic diversity

Analysis of microsatellite data for rainbow trout (reported in Consuegra et al. 2011) indicated that there were no outliers with either lositan or bayescan that could be indicative of divergent selection (Table S3b). In general, rainbow trout showed similar levels of heterozygosity (*H*_*o*_) and allelic richness (AR) than brown trout (as evidenced by overlapping 95CIs, Fig. 2). Estimates of effective population size (*N*e) revealed small population sizes, similar to those of brown trout (*N*_e_ < 50; Table 1), but rainbow trout generally exhibited more admixture and weaker differentiation than brown trout in Chile (Fig. 3), particularly in those populations most affected by secondary releases from aquaculture (Fig. 4, high admixture). There was no correlation between spatial and genetic distance (*z* = −14 055.14, *r* = 0.034, *P* = 0.361).

#### Phenotypic differentiation

Rainbow trout ranged between 50 and 245 mm in fork length, and the minimal adequate linear mixed-effects model that explained variation in scale intercirculi spacing included population (*F*_6,122_ = 16.15, *P* < 0.001) and age (*F*_1,122_ = 9.34, *P =* 0.003) as fixed factors and an interaction between population and individual fork length (*F*_6,122_ = 2.62, *P =* 0.02; Figure S1A). As with brown trout, rainbow trout populations also showed significant differences in the three other growth-related traits examined, that is, condition factor (*F*_5, 116_ = 35.81, *P* < 0.001), number of growth circuli deposited during the first freshwater year (*F*_6, 131_ = 8.43, *P* < 0.001), and scale size at the end of the first year (*F*_6, 131_ = 5.57, *P* < 0.001).

#### *P*_ST_/*F*_ST_ comparisons

**Figure 5:**
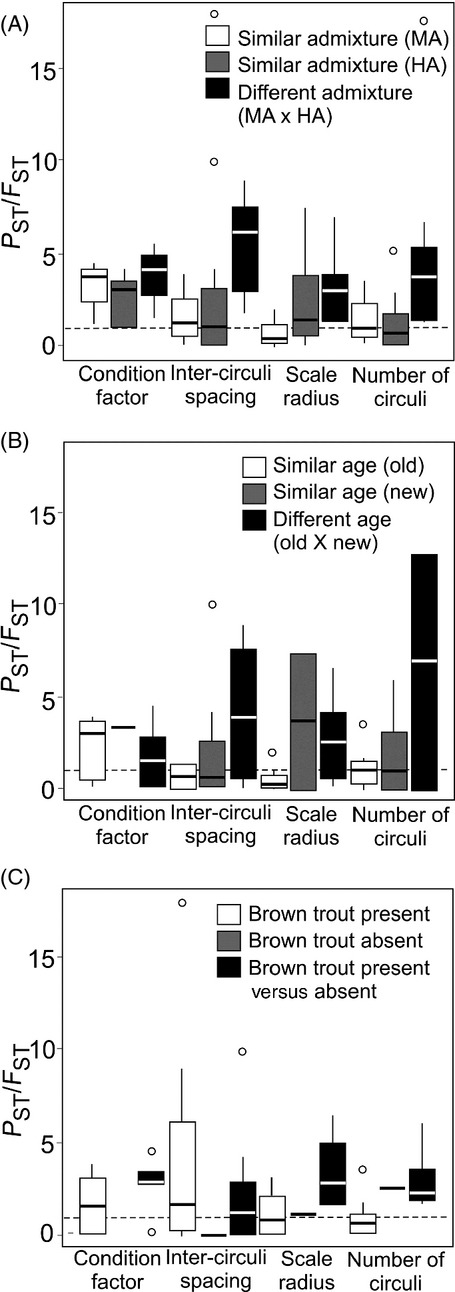
*P*_ST_/*F*_ST_ for rainbow trout having different (A) levels of admixture (MA = moderate admixture versus HA = high admixture), (B) population age (residence time, old versus new) and (C) coexistence with brown trout (present versus absent). Dashed line represents neutral expectation (*P*_ST_/*F*_ST_ = 1).

*P*_ST_/*F*_ST_ was significantly higher than the neutral expectation for all traits and for most values of *c*, indicating that the contribution of environmental effects to phenotypic variance was minimal (Table 3; Figure S2B). Pairwise *P*_ST_/*F*_ST_ comparisons between populations with similar levels of admixture were not significantly different from random expectations at any trait except for condition factor (Fig. 5A). In contrast, *P*_ST_/*F*_ST_ comparisons between populations with different levels of admixture were significantly different from 1 at all traits examined (Fig. 5A). None of the pairwise *P*_ST_/*F*_ST_ comparisons differed significantly from 1 when populations of the same or different relative population age were compared (Fig. 5B), suggesting that residence time did not have a significant effect on phenotypic divergence. When comparisons were made between populations living in sympatry or in allopatry with brown trout, pairwise *P*_ST_/*F*_ST_ values were significantly different than 1 for all traits except intercirculi spacing during the first winter (Fig. 5C), suggesting that coexistence with brown trout may have affected the adaptive divergence of rainbow trout populations. In addition, interspecific competition explained a significant amount of molecular variation (Table 2). As with brown trout, geographical distance between rainbow trout populations was unrelated to *P*_ST_, *F*_ST,_ or *P*_ST_/*F*_ST_ (Table S5).

## Discussion

Divergence among invaders should increase with both residence time and secondary releases because older populations would have had more time to adapt to novel conditions and new alleles can extend the window of opportunity for invasions to succeed (Dlugosch and Parker 2008; Crawford and Whitney 2010; Dainese and Poldini 2012). We employed *P*_ST_/*F*_ST_ comparisons to assess such predictions in two invasive salmonids, rainbow trout and brown trout, screened for microsatellite DNA variation and growth-related traits in rivers of Chilean Patagonia and the Falkland Islands. Using *P*_ST_ as a phenotypic analogue for *Q*_ST_ has limitations (Pujol et al. 2008) because environmental effects may introduce errors in the estimation of variance components, underestimating the within-population variance and overestimating the among-population variance (Leinonen et al. 2013). Despite this caveat, a meta-analysis has shown that *P*_ST_ and *Q*_ST_ estimates do not differ systematically (Leinonen et al. 2008), and *P*_ST_ still provides one of the few options available for studying phenotypic divergence in natural populations in the wild, when common garden experiments are not normally possible (Keller and Taylor 2008). Hence, while we acknowledge the limitations of *P*_ST,_ we believe that a comparative analysis of *P*_ST/_*F*_ST_ across species and traits might be useful and shed light on the adaptation of invasive species to novel environments, a largely unexplored aspect of *Q*_ST_/*F*_ST_ studies (reviewed by Leinonen et al. 2013).

We tested for the effects of residence time by examining divergence of trout populations of different ages. In the case of brown trout, population age was inferred from the presence or absence of native galaxiid fishes – the absence of galaxiid being indicative of older invasions (Young et al. 2010), and from historical records – brown trout populations being generally older in Chile than in the Falklands (Arrowsmith and Pentelow 1965). In the case of rainbow trout, we inferred population age from genetic similarity to farm fish, a high similarity being typical of recent, aquaculture-driven invasions (Consuegra et al. 2011). Contrary to our expectations, we did not find significant differences in genetic diversity, effective population size, or signatures of expansion between brown trout populations with different residence times. Phenotypic divergence did not increase with geographical location, and population age did not make a significant contribution to the extent of molecular variation in any of the two trout species. Our results, therefore, do not support the contention that older trout populations are more differentiated than younger ones in this area. This suggests that other factors, such as secondary releases (or genetic drift), may have been more instrumental than residence time in maintaining genetic diversity and in generating population differentiation, as suggested for other organisms (Ellstrand and Schierenbeck 2000; Bossdorf et al. 2005; Crawford and Whitney 2010).

A higher level of admixture was detected in rainbow trout than in brown trout, and unlike brown trout, where no evidence of adaptive divergence was found with respect to population age or broad geographical location, *P*_ST_/*F*_ST_ comparisons in rainbow trout were consistent with divergent selection at all phenotypic traits examined, albeit only between populations showing different levels of admixture. Unlike brown trout, which is not commercially farmed to any extent in the area, rainbow trout is extensively farmed in Chilean Patagonia, and this has resulted in a large influx of farm escapees from many different sources interbreeding with existing, naturalized populations (Consuegra et al. 2011). Secondary releases of rainbow trout could have facilitated invasion not only by restoring or increasing genetic and phenotypic diversity, but also because reintroduced populations have often overcome the establishment phase of the invasion process, a phase which is often accompanied by demographic and genetic bottlenecks (Novak and Mack 2005). In addition, it is also possible that hybridization between rainbow trout escapees and naturalized individuals may have increased standing genetic variation or resulted in heterosis (i.e., hybrids with superior fitness; Fraser 2008) at least during the first generations. The observed increase in *P*_ST_/*F*_ST_ ratios could have resulted from genetic introgression with farm fish, as farm fish are likely to have been selected for fast growth.

Whatever the precise reasons for the increased *P*_ST_/*F*_ST_ values observed among rainbow trout, we failed to find similar evidence in brown trout, which are not generally affected by secondary releases in our study. The only exception was Estancia Brook, a population in East Falkland which had an unusually high number of private alleles (*P*_A_ = 20), and in which our assignment grouped with Chilean populations of presumably older age. Such a result is consistent with what is known about multiple origins of brown trout in the Falklands: an initial introduction likely from German origin (Estay et al. 2004) shipped via Chile in 1936–1947, followed by more recent and extensive introductions from Britain during 1948–1962 (Arrowsmith and Pentelow 1965; Stewart 1973). Thus, it appears that brown trout in some parts of the Falklands, as rainbow trout affected by aquaculture escapees in Chile, may have diverged due to secondary releases, not due to residence time. A similar situation appears to exist in the Kerguelen Islands where brown trout of mixed origins has shown rapid genetic differentiation despite a very short residence time (<20 years, Ayllon et al. 2006), indicating that divergence can occur rapidly when introductions are aided by secondary releases. The only brown trout population that showed clear signatures of population expansion in our study was one of the youngest populations in the Falklands (Sarnys Creek). None of the presumably older populations in Chile showed genetic evidence of population expansion, suggesting that residency time may not be a good predictor of colonization potential in this area.

In their native range, brown trout populations tend to be highly structured and even populations in nearby streams often show significant differentiation conductive of local adaptations (Bernatchez 2001; Carlsson et al. 2005; Skaala 2006); recent studies suggest that genetic and phenotypic divergence can result from environmental variation (Keller et al. 2011, 2012; Stelkens et al. 2012). Our results reveal the existence of high population structuring with no evidence of IBD also among much younger populations in Chile and the Falkland Islands, as indicated previously by studies of allozyme variation (Faundez et al. 1997; Colihueque et al. 2003). Freshwater residence would result in low gene flow and high population structuring. We only identified 2% of our trout (all in the Falklands) as anadromous fish (sea trout) based on scale growth patterns, although the body size of our samples (93% were below 300 mm fork length) must have limited our capacity to detect migrants. Despite this caveat, limited anadromy among brown trout is consistent with recent studies in the area (Young et al. 2010) and also with a presumed nonmigratory life history of many of the donor trout populations introduced into Chile (Faundez et al. 1997) and the Falkland Islands (McDowall et al. 2001).

The capacity to grow quickly has been flagged as an important determinant of invasion success (Townsend 2003) because prey–predator interactions in freshwater are strongly mediated by size differences and fast growth enables fish invaders to reproduce quickly and become piscivorous sooner. We found significant population differences in both brown trout and rainbow trout in three of the four growth traits examined, suggesting that populations were growing at different rates during their first year. Yet, none of these growth-related traits showed evidence of adaptive divergence with respect to population age in any species, perhaps because populations were too young to have developed local adaptations or because there was no divergent selection for the traits under study. Size-related traits tend to have relatively high heritability in salmonids (Garcia de Leaniz et al. 2007) and although these may be expected to respond rapidly to novel selective pressures during invasions, they may not be tightly correlated with fitness (Merilä and Sheldon 1999). We used a *h*^2^ of 0.25 for body size and growth rate in freshwater based on salmonid studies carried out mostly in captivity (Garcia de Leaniz et al. 2007; Carlson and Seamons 2008), which may not necessarily be relevant in the field (Hoffmann 2000). However, the same or very similar heritability estimates have also been obtained in salmonid field studies (e.g., brook trout = 0.25, Letcher et al. 2011; Atlantic salmon = 0.27, Garant et al. 2003). Given that heritability for body size appears to be 34% of the repeatability estimate in the field (Letcher et al. 2011), this would yield a *h*^2^ of 0.31 for rainbow trout and 0.26 for brown trout in our study, not markedly different from the value of 0.25 used in our *P*_ST_/*F*_ST_ simulations. Future studies might benefit from measuring additional traits, ideally under common garden conditions, and to compare ancestral and invasive lineages in order to tease apart the effects of founder effects, local adaptations, and phenotypic plasticity (Leinonen et al. 2008), as shown recently for brown trout in North America (Westley et al. 2013).

Rainbow trout exhibits a wider geographical range than brown trout in Chile (Young et al. 2010), and its expansion seems to have been limited chiefly by habitat connectivity and temperature (Habit et al. 2012). Brown trout and rainbow trout do not naturally coexist in their native ranges, and laboratory and field studies have shown that survival and habitat selection by brown trout is negatively affected by the presence of rainbow trout under sympatric conditions (Blanchet et al. 2007). Pairwise *P*_ST_/*F*_ST_ comparisons between rainbow trout living in sympatry or in allopatry with brown trout were significantly higher than 1 in three of the four growth-related traits examined, suggesting that competitive interactions may have resulted in adaptive divergence of rainbow trout when these two invaders were translocated together with the southern hemisphere.

In summary, residence time did not explain well the observed patterns of genetic and phenotypic divergence among invasive trout in our study, as some of the youngest populations were also the most genetically diverse ones. Although the conditions necessary for adaptive divergence appear to exist (i.e., high genetic variability, high population structure, and habitat heterogeneity at the relevant spatial scales – see Young et al. 2010; Vanhaecke et al. 2012a), we did not find significant evidence of adaptive divergence in growth-related traits with respect to population age. Instead, our results highlight a potential role for secondary releases in generating divergence of invasive salmonids in the area, particularly for rainbow trout. We also found that coexistence with brown trout made a significant contribution to molecular variation in rainbow trout, and some evidence to suggest that phenotypic divergence in rainbow trout may have also increased in rivers where the two trout invaders coexist. Such knowledge is important for understanding and predicting the effects of fish invasions because the diversity of fish invaders could affect their impact upon native fish fauna (Blanchet et al. 2007; but see Young et al. 2009). Previous studies have examined adaptive differentiation in translocated fishes (e.g., Hendry et al. 2000; Unwin et al. 2000; Koskinen et al. 2002; Kinnison et al. 2008), but these have usually dealt with single species and/or single systems, making it difficult to test predictions derived from competing hypotheses. By considering simultaneously two fish species translocated together into a number of common and different river systems, our study of *P*_ST_/*F*_ST_ comparisons provides insights into the nature of diversifying forces acting during fish invasions.

## Data archiving statement

Raw experimental data for this study are available from FigShare: Garcia de Leaniz, Carlos; Monzón-Argüello, C.; Consuegra, S.; Gajardo, G.; Marco-Rius, F.; Fowler, D.M.; DeFaveri, J. (2014): Genetic variation brown trout Chile & Falklands. figshare. http://dx.doi.org/10.6084/m9.figshare.1067046.

## Supporting Information

**Figure S1.** Individual growth curves, represented as cumulative scale growth profiles (mm) at each growth circulus, among invasive brown trout (*n*: 5) and rainbow trout (*n*: 7) populations.

**Figure S2.** Relationship between observed (solid line) and neutral (dashed line) *P*_ST_/*F*_ST_, for four phenotypic traits of (a) brown trout and (b) rainbow trout.

**Table S1.** Strains of rainbow trout and brown trout introduced in Chile and the Falkland Islands.

**Table S2**. Summary genetic statistics for brown trout considering (a) global sample (over all populations) and (b) population-specific.

**Table S3**. Results of bayescan and lositan programs for outlier detection in (a) brown trout and (b) rainbow trout.

**Table S4.** (a) Differentiation of brown trout populations expressed as (a) pairwise *F*_*ST*_ comparisons (below diagonal) and *D*_est_ (above diagonal) among Chilean and Falklands populations and (b) as pairwise *F*_ST_ estimated from neutral (below diagonal) and gene-linked (above diagonal) markers.

**Table S5.** Correlations between *P*_ST_, *F*_ST_, *P*_ST_/*F*_ST_ and geographical distance.

## References

[b1] Alpert P, Bone E, Holzapfel C (2000). Invasiveness, invasibility and the role of environmental stress in the spread of non-native plants. Perspectives in Plant Ecology, Evolution and Systematics.

[b2] Antao T, Lopes A, Lopes R, Beja-Pereira A, Luikart G (2008). LOSITAN: a workbench to detect molecular adaptation based on a Fst-outlier method. BMC Bioinformatics.

[b3] Arismendi I, Soto D, Penaluna B, Jara C, Leal C, León-Muñoz J (2009). Aquaculture, non-native salmonid invasions and associated declines of native fishes in Northern Patagonian lakes. Freshwater Biology.

[b4] Arrowsmith E, Pentelow F (1965). The introduction of trout and salmon to the Falkland Islands. Salmon and Trout Magazine.

[b5] Ayllon F, Davaine P, Beall E, Garcia-Vazquez E (2006). Dispersal and rapid evolution in brown trout colonizing virgin Subantarctic ecosystems. Journal of Evolutionary Biology.

[b6] Barrett RDH, Schluter D (2008). Adaptation from standing genetic variation. Trends in Ecology and Evolution.

[b7] Basulto S (2003). El Largo Viaje de los Salmones: una Crónica Olvidada. Propagación y Cultivo de Especies Acuáticas en Chile.

[b8] Bernatchez L (2001). The evolutionary history of brown trout (*Salmo trutta* L.) inferred from phylogeographic, nested clade, and mismatch analyses of mitochondrial DNA variation. Evolution.

[b9] Bilgin R (2007). Kgtests: a simple Excel Macro program to detect signatures of population expansion using microsatellites. Molecular Ecology Notes.

[b10] Blackwell BG, Brown ML, Willis DW (2000). Relative weight (Wr) status and current use in fisheries assessment and management. Reviews in Fisheries Science.

[b11] Blanchet S, Loot G, Grenouillet G, Brosse S (2007). Competitive interactions between native and exotic salmonids: a combined field and laboratory demonstration. Ecology of Freshwater Fish.

[b12] Bossdorf O, Auge H, Lafuma L, Rogers WE, Siemann E, Prati D (2005). Phenotypic and genetic differentiation between native and introduced plant populations. Oecologia.

[b13] Brommer JE (2011). Whither *P*_ST_? The approximation of *Q*_ST_ by *P*_ST_ in evolutionary and conservation biology. Journal of Evolutionary Biology.

[b14] Cairney M, Taggart JB, Hoyheim B (2000). Characterization of microsatellite and minisatellite loci in Atlantic salmon (*Salmo salar* L.) and cross-species amplification in other salmonids. Molecular Ecology.

[b152] Carlson SM, Seamons TR (2008). A review of quantitative genetic components of fitness in salmonids: implications for adaptation to future change. Evolutionary Applications.

[b15] Carlsson J, Olsen K, Nilsson J, Øverli Ø, Stabell O (2005). Microsatellites reveal fine-scale genetic structure in stream-living brown trout. Journal of Fish Biology.

[b16] Chen C, Durand E, Forbes F, François O (2007). Bayesian clustering algorithms ascertaining spatialm population structure: a new computer program and a comparison study. Molecular Ecology Notes.

[b17] Colihueque N, Iturra P, Estay F, Díaz NF (2001). Diploid chromosome number variations and sex chromosome polymorphism in five cultured strains of rainbow trout (*Oncorhynchus mykiss*. Aquaculture.

[b18] Colihueque N, Vergara N, Parraguez M (2003). Genetic characterization of naturalized populations of brown trout *Salmo trutta* L. in southern Chile using allozyme and microsatellite markers. Aquaculture Research.

[b19] Consuegra S, Phillips N, Gajardo G, Garcia de Leaniz C (2011). Winning the invasion roulette: escapes from fish farms increase admixture and facilitate establishment of non-native rainbow trout. Evolutionary Applications.

[b20] Cornuet JM, Luikart G (1996). Description and power analysis of two tests for detecting recent population bottlenecks from allele frequency data. Genetics.

[b21] Correa C, Hendry AP (2012). Invasive salmonids and lake order interact in the decline of puye grande *Galaxias platei* in western Patagonia lakes. Ecological Applications.

[b22] Crawford NG (2010). smogd: software for the measurement of genetic diversity. Molecular Ecology Resources.

[b23] Crawford SS, Muir AM (2008). Global introductions of salmon and trout in the genus *Oncorhynchus*: 1870-2007. Reviews in Fish Biology and Fisheries.

[b24] Crawford KM, Whitney KD (2010). Population genetic diversity influences colonization success. Molecular Ecology.

[b25] Dainese M, Poldini L (2012). Does residence time affect responses of alien species richness to environmental and spatial processes?. NeoBiota.

[b26] Dlugosch KM, Parker IM (2008). Founding events in species invasions: genetic variation, adaptive evolution, and the role of multiple introductions. Molecular Ecology.

[b27] Ellstrand NC, Schierenbeck KA (2000). Hybridization as a stimulus for the evolution of invasiveness in plants?. Proceedings of the National Academy of Sciences USA.

[b28] Estay FJ, Noriega R, Ureta JP, Martin W, Colihueque N (2004). Reproductive performance of cultured brown trout (*Salmo trutta* L.) in Chile. Aquaculture Research.

[b29] Estoup A, Presa P, Krieg F, Vaiman D, Guyomard R (1993). CT)(N) and (GT)(N) microsatellites – a new class of genetic-markers for *Salmo trutta* L (Brown trout). Heredity (Edinb).

[b30] Evanno G, Regnaut S, Goudet J (2005). Detecting the number of clusters of individuals using the softwarestructure: a simulation study. Molecular Ecology.

[b31] Excoffier L, Lischer HEL (2010). Arlequin suite ver 3.5: a new series of programs to perform population genetics analyses under Linux and Windows. Molecular Ecology Resources.

[b32] Facon B, Genton BJ, Shykoff J, Jarne P, Estoup A, David P (2006). A general eco-evolutionary framework for understanding bioinvasions. Trends in Ecology & Evolution.

[b33] Faundez V, Blanco G, Vázquez E, Sánchez J (1997). Allozyme variability in brown trout *Salmo trutta* in Chile. Freshwater Biology.

[b34] Foll M, Gaggiotti O (2008). A genome-scan method to identify selected loci appropriate for both dominant and codominant markers: a Bayesian perspective. Genetics.

[b35] Fraser DJ (2008). How well can captive breeding programs conserve biodiversity? A review of salmonids. Evolutionary Applications.

[b36] Gajardo G, Laikre L (2003). Chilean Aquaculture boom is based on exotic salmon resources: a conservation paradox. Conservation Biology.

[b154] Garant D, Dodson JJ, Bernatchez L (2003). Differential reproductive success and heritability of alternative reproductive tactics in wild atlantic salmon (*Salmo salar* L.). Evolution.

[b37] Garcia de Leaniz C, Fleming IA, Einum S, Verspoor E, Jordan WC, Consuegra S, Aubin-Horth N (2007). A critical review of adaptive genetic variation in Atlantic salmon: implications for conservation. Biological Reviews.

[b38] Garcia de Leaniz C, Gajardo G, Consuegra S (2010). From best to pest: changing perspectives on the impact of exotic salmonids in the Southern Hemisphere. Systematics and Biodiversity.

[b39] Belkhir K, Borsa P, Chikhi L, Raufaste N, Bonhomme F (2001). GENETIX 4.02, logiciel sous Windows TM pour la génétique des populations: Laboratoire Génome, Populations, Interactions, CNRS UMR.

[b40] Goudet J (1995). FSTAT (Version 1.2): a computer program to calculate F-statistics. Journal of Heredity.

[b41] Goudet J (2005). HIERFSTAT, a package for R to compute and test hierarchical F-statistics. Molecular Ecology Notes.

[b42] Grimholt U, Hordvik I, Fosse VM, Olsaker I, Endresen C, Lie Ø (1993). Molecular cloning of major histocompatibility complex class I cDNAs from Atlantic salmon (*Salmo salar*. Immunogenetics.

[b43] Grimholt U, Drablos F, Jorgensen SM, Hoyheim B, Stet RJ (2002). The major histocompatibility class I locus in Atlantic salmon (*Salmo salar* L.): polymorphism, linkage analysis and protein modelling. Immunogenetics.

[b44] Habit E, Gonzalez J, Ruzzante DE, Walde SJ (2012). Native and introduced fish species richness in Chilean Patagonian lakes: inferences on invasion mechanisms using salmonid-free lakes. Diversity and Distributions.

[b45] Hendry AP, Wenburg JK, Bentzen P, Volk EC, Quinn TP (2000). Rapid evolution of reproductive isolation in the wild: evidence from introduced salmon. Science.

[b155] Hoffmann AA, Mousseau TA, Sinervo B, Endler J (2000). Laboratory and field heritabilities. Some lessons from *Drosophila*. Adaptive Genetic Variation in the Wild.

[b46] Holand AM, Jensen H, Tufto J, Moe R (2011). Does selection or genetic drift explain geographic differentiation of morphological characters in house sparrows *Passer domesticus*. Genetics Research.

[b47] Jones OR, Wang J (2010). COLONY: a program for parentage and sibship inference from multilocus genotype data. Molecular Ecology Resources.

[b48] Jost L (2008). GST and its relatives do not measure differentiation. Molecular Ecology.

[b49] Keller SR, Taylor DR (2008). History, chance and adaptation during biological invasion: separating stochastic phenotypic evolution from response to selection. Ecology Letters.

[b50] Keller I, Taverna A, Seehausen O (2011). Evidence of neutral and adaptive genetic divergence between European trout populations sampled along altitudinal gradients. Molecular Ecology.

[b51] Keller I, Schuler J, Bezault E, Seehausen O (2012). Parallel divergent adaptation along replicated altitudinal gradients in Alpine trout. BMC Evolutionary Biology.

[b52] King TL, Eackles MS, Letcher BH (2005). Microsatellite DNA markers for the study of Atlantic salmon (*Salmo salar*) kinship, population structure, and mixed-fishery analyses. Molecular Ecology Notes.

[b53] Kinnison MT, Unwin MJ, Quinn TP (2008). Eco-evolutionary vs. habitat contributions to invasion in salmon: experimental evaluation in the wild. Molecular Ecology.

[b54] Kolar CS, Lodge DM (2001). Progress in invasion biology: predicting invaders. Trends in Ecology and Evolution.

[b55] Kolar CS, Lodge DM (2002). Ecological predictions and risk assessment for alien fishes in North America. Science.

[b56] Kolbe JJ, Glor RE, Rodriguez Schettino L, Lara AC, Larson A, Losos JB (2004). Genetic variation increases during biological invasion by a Cuban lizard. Nature.

[b57] Koskinen MT, Haugen TO, Primmer CR (2002). Contemporary fisherian life-history evolution in small salmonid populations. Nature.

[b58] Kowarik I (2003). Human agency in biological invasions: secondary releases foster naturalisation and population expansion of alien plant species. Biological Invasions.

[b59] Lande R (2009). Adaptation to an extraordinary environment by evolution of phenotypic plasticity and genetic assimilation. Journal of Evolutionary Biology.

[b60] Lavergne S, Molofsky J (2007). Increased genetic variation and evolutionary potential drive the success of an invasive grass. Proceedings of the National Academy of Sciences USA.

[b61] Lee CE (2002). Evolutionary genetics of invasive species. Trends in Ecology and Evoution.

[b62] Leinonen T, O'Hara RB, Cano JM, Merilä J (2008). Comparative studies of quantitative trait and neutral marker divergence: a meta-analysis. Journal of Evolutionary Biology.

[b63] Leinonen T, McCairns RS, O'Hara RB, Merilä J (2013). *Q*_ST_–*F*_ST_ comparisons: evolutionary and ecological insights from genomic heterogeneity. Nature Reviews Genetics.

[b156] Letcher BH, Coombs JA, Nislow KH (2011). Maintenance of phenotypic variation: repeatability, heritability and size-dependent processes in a wild brook trout population. Evolutionary Applications.

[b64] Lhorente JP (2011). http://www.biomar.com/Countries/Chile/Eventos1/Lhorente.pdf.

[b65] Lockwood JL, Cassey P, Blackburn T (2005). The role of propagule pressure in explaining species invasions. Trends in Ecology & Evolution.

[b66] Lonsdale WM (1999). Global patterns of plant invasions and the concept of invasibility. Ecology.

[b67] Lowe S, Browne M, Boudjelas S, De Poorter M (2000).

[b68] MacCrimmon HR (1971). World distribution of rainbow trout (*Salmo gairdneri*. Journal of the Fisheries Board of Canada.

[b69] MacCrimmon HR, Marshall T (1968). World distribution of brown trout, *Salmo trutta*. Journal of the Fisheries Board of Canada.

[b70] Marchetti MP, Moyle PB, Levine R (2004). Invasive species profiling? Exploring the characteristics of non-native fishes across invasion stages in California. Freshwater Biology.

[b71] Marco-Rius F, Caballero P, Morán P, Garcia de Leaniz C (2012). And the last shall be first: heterochrony and compensatory marine growth in sea trout (*Salmo trutta*. PLoS One.

[b72] Marco-Rius F, Caballero P, Morán P, Garcia de Leaniz C (2013). Mixed-effects modelling of scale growth profiles predicts the occurrence of early and late fish migrants. PLoS One.

[b73] McDowall RM (2006). Crying wolf, crying foul, or crying shame: alien salmonids and a biodiversity crisis in the southern cool-temperate galaxioid fishes?. Reviews in Fish Biology and Fisheries.

[b74] McDowall R, Allibone R, Chadderton W (2001). Issues for the conservation and management of Falkland Islands freshwater fishes. Aquatic Conservation: Marine and Freshwater Ecosystems.

[b75] Merilä J, Crnokrak P (2001). Comparison of genetic differentiation at marker loci and quantitative traits. Journal of Evolutionary Biology.

[b76] Merilä J, Sheldon B (1999). Genetic architecture of fitness and nonfitness traits: empirical patterns and development of ideas. Heredity (Edinb).

[b77] Mobley KB, Lussetti D, Johansson F, Englund G, Bokma F (2011). Morphological and genetic divergence in Swedish postglacial stickleback (*Pungitius pungitius*) populations. BMC Evolutionary Biology.

[b78] Monzón-Argüello C, Garcia de Leaniz C, Gajardo G, Consuegra S (2013). Less can be more: loss of MHC functional diversity can reflect adaptation to novel conditions during fish invasions. Ecology and Evolution.

[b79] Nei M (1987). Molecular Evolutionary Genetics.

[b80] Nei M, Maruyama T, Chakraborty R (1975). The Bottleneck effect and genetic variability in populations. Evolution.

[b81] Novak SJ, Sax DF, Stachowicz JJ, Gaines SD, Mack RN (2005). Genetic bottlenecks in alien plant species: influence of mating systems and introduction dynamics. Species Invasions: Insights into Ecology, Evolution, and Biogeography.

[b82] Olafsson K, Hjorleifsdottir S, Pampoulie C, Hreggvidsson GO, Gudjonsson S (2010). Novel set of multiplex assays (SalPrint15) for efficient analysis of 15 microsatellite loci of contemporary samples of the Atlantic salmon (*Salmo salar*. Molecular Ecology Resources.

[b83] O'Reilly P, Hamilton L, McConnell S, Wright J (1996). Rapid analysis of genetic variation in Atlantic salmon (*Salmo salar*) by PCR multiplexing of dinucleotide and tetranucleotide microsatellites. Canadian Journal of Fisheries and Aquatic Sciences.

[b84] Perrings C, Dehnen-Schmutz K, Touza J, Williamson M (2005). How to manage biological invasions under globalization. Trends in Ecology and Evolution.

[b85] Pinheiro JC, Bates DM (2009). Mixed-Effects Models in S and S-Plus.

[b86] Prentis PJ, Wilson JRU, Dormontt EE, Richardson DM, Lowe AJ (2008). Adaptive evolution in invasive species. Trends in Plant Science.

[b87] Pritchard JK, Stephens M, Donnelly P (2000). Inference of population structure using multilocus genotype data. Genetics.

[b88] Pujol B, Wilson A, Ross R, Pannell J (2008). Are QST–FST comparisons for natural populations meaningful?. Molecular Ecology.

[b89] Pyšek P, Inderjit S, Jarošík V (2005). Residence time determines the distribution of alien plants. Invasive Plants: Ecological and Agricultural Aspects.

[b158] R Development Core Team (2008). R: A Language and Environment for Statistical Computing.

[b90] Reich DE, Feldman MW, Goldstein DB (1999). Statistical properties of two teststhat use multilocus data sets to detect population expansions. Molecular Biology and Evolution.

[b91] Reznick DN, Ghalambor CK (2001). The population ecology of contemporary adaptations: what empirical studies reveal about the conditions that promote adaptive evolution. Genetica.

[b92] Richards CL, Bossdorf O, Muth NZ, Gurevitch J, Pigliucci M (2006). Jack of all trades, master of some? On the role of phenotypic plasticity in plant invasions. Ecology Letters.

[b93] Schröder V, Garcia de Leaniz C (2011). Discrimination between farmed and free-living invasive salmonids in Chilean Patagonia using stable isotope analysis. Biological Invasions.

[b94] Skaala Ø (2006). Genetic population structure of Norwegian brown trout. Journal of Fish Biology.

[b95] Slettan A, Olsaker I, Lie Ø (1995). Atlantic salmon, *Salmo salar*, microsatellites at the SSOSL25, SSOSL85, SSOSL311, SSOSL417 loci. Animal Genetics.

[b96] Spiegelhalter DJ, Best NG, Carlin BP, Van Der Linde A (2002). Bayesian measures of model complexity and fit. Journal of the Royal Statistical Society: Series B (Statistical Methodology).

[b97] Spitze K (1993). Population structure in *Daphnia obtusa*: quantitative genetic and allozymic variation. Genetics.

[b98] Stelkens RB, Jaffuel G, Escher M, Wedekind C (2012). Genetic and phenotypic population divergence on a microgeographic scale in brown trout. Molecular Ecology.

[b99] Stewart L (1973). The Fisheries in the Falkland Islands.

[b100] Tallmon DA, Koyuk A, Luikart G, Beaumont MA (2008). ONeSAMP: a program to estimate effective population size using approximate Bayesian computation. Molecular Ecology Resources.

[b101] Thompson JN (1998). Rapid evolution as an ecological process. Trends in Ecology and Evolution.

[b102] Townsend CR (2003). Individual, population, community, and ecosystem consequences of a fish invader in New Zealand streams. Conservation Biology.

[b103] Unwin MJ, Quinn TP, Kinnison MT, Boustead NC (2000). Divergence in juvenile growth and life history in two recently colonized and partially isolated chinook salmon populations. Journal of Fish Biology.

[b106] Van Oosterhout C, Hutchinson WF, Wills DPM, Shipley P (2004). MICRO-CHECKER: software for identifying and correcting genotyping errors in microsatellite data. Molecular Ecology Notes.

[b107] Vanhaecke D, Garcia de Leaniz C, Gajardo G, Thomas CJ, Consuegra S (2012a). Metapopulation dynamics of a diadromous galaxiid fish and potential effects of salmonid aquaculture. Freshwater Biology.

[b108] Vanhaecke D, Garcia de Leaniz C, Gajardo G, Young K, Sanzana J, Orellana G, Fowler D (2012b). DNA barcoding and microsatellites help species delimitation and hybrid identification in endangered galaxiid fishes. PLoS One.

[b109] Vasemagi A, Nilsson J, Primmer CR (2005). Expressed sequence tag-linked microsatellites as a source of gene-associated polymorphisms for detecting signatures of divergent selection in Atlantic salmon (*Salmo salar* L.). Molecular Biology and Evolution.

[b110] Westley PAH, Ward EJ, Fleming IA (2013). Fine-scale local adaptation in an invasive freshwater fish has evolved in contemporary time. Proceedings of the Royal Society B: Biological Sciences.

[b111] Whitlock MC, Guillaume F (2009). Testing for spatially divergent selection: comparing *Q*_ST_ to *F*_ST_. Genetics.

[b112] Wilson JRU, Richardson DM, Rouget M, Procheş Ş, Amis MA, Henderson L, Thuiller W (2007). Residence time and potential range: crucial considerations in modelling plant invasions. Diversity and Distributions.

[b113] Wolak ME, Fairbairn DJ, Paulsen YR (2012). Guidelines for estimating repeatability. Methods in Ecology and Evolution.

[b114] Young KA, Stephenson J, Terreau A, Thailly AF, Gajardo G, Garcia de Leaniz C (2009). The diversity of juvenile salmonids does not affect their competitive impact on a native galaxiid. Biological Invasions.

[b115] Young KA, Dunham JB, Stephenson JF, Terreau A, Thailly AF, Gajardo G, Garcia de Leaniz C (2010). A trial of two trouts: comparing the impacts of rainbow and brown trout on a native galaxiid. Animal Conservation.

