# Supplementary figures and images for: Contrasting patterns of genetic and phenotypic differentiation in two invasive salmonids in the southern hemisphere
Source: Evol Appl. 2014 Jul 23;7(8):921–36. doi: 10.1111/eva.12188 (PMC4211722; doi:10.1111/eva.12188)

**(A) Brown Trout**

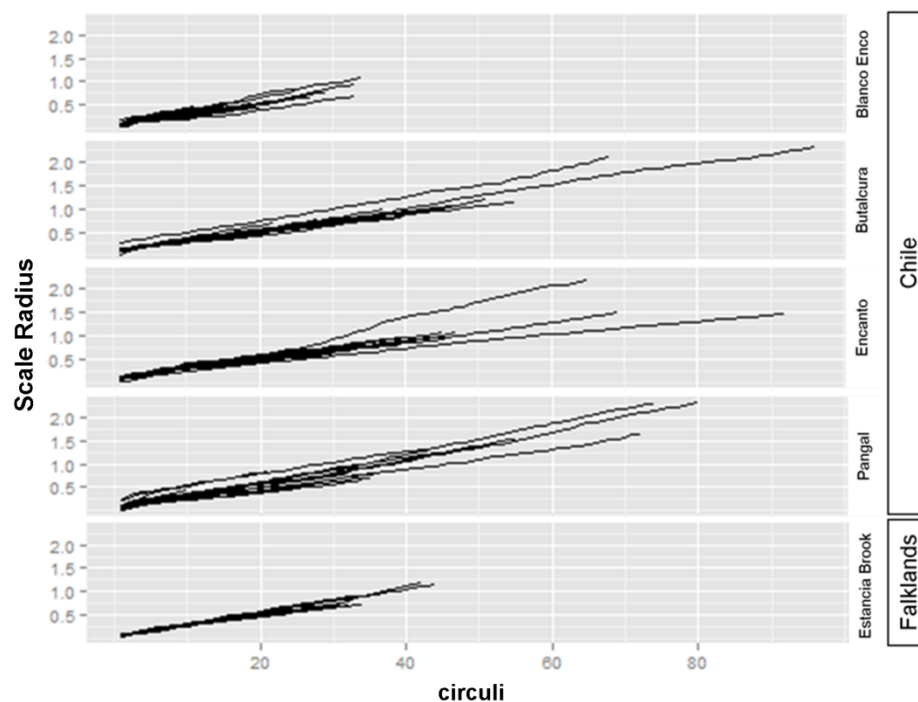

**(B) Rainbow Trout**

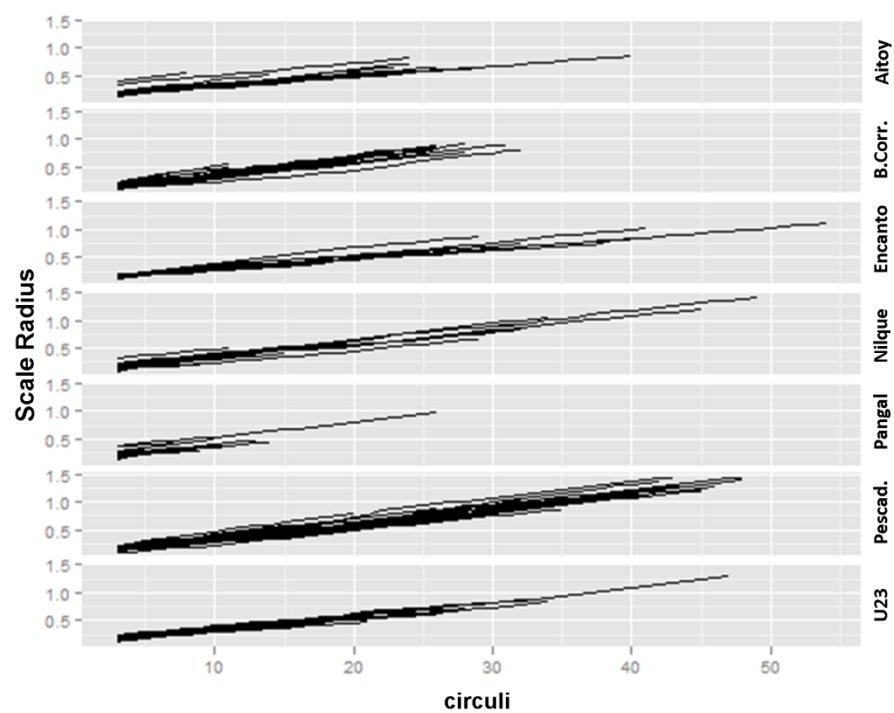

Supplement: Supplementary file 1 — Figure S1. Individual growth curves, represented as cumulative scale growth profiles (mm) at each growth circulus, among invasive brown trout (n: 5) and rainbow trout (n: 7) populations. [file eva0007-0921-sd1.pdf]

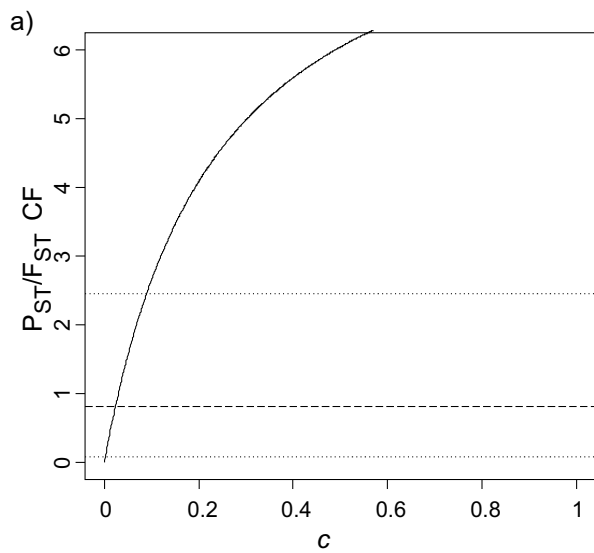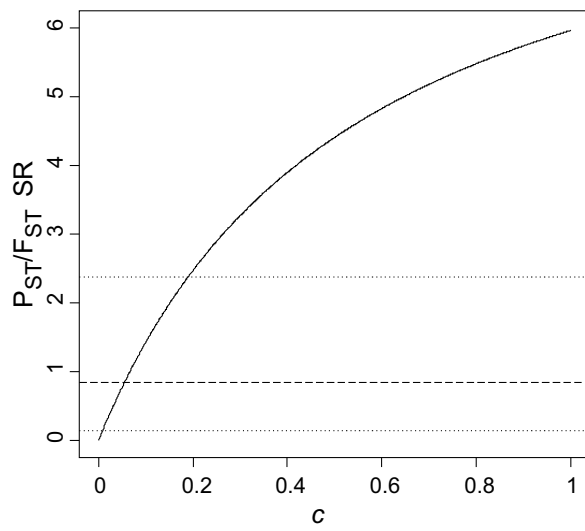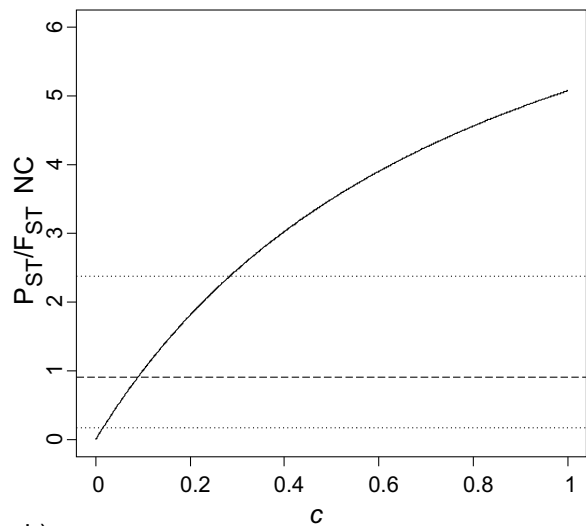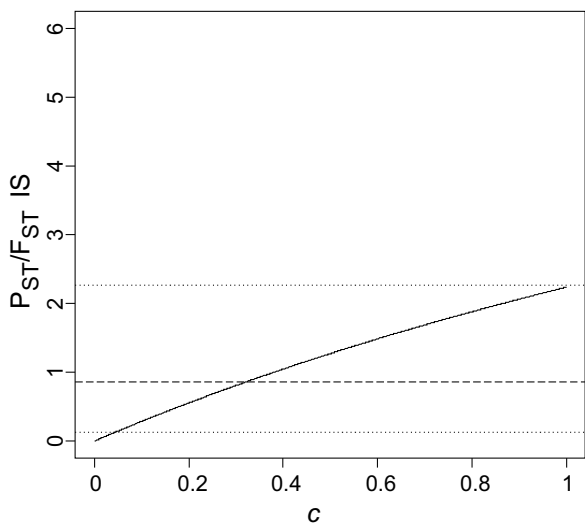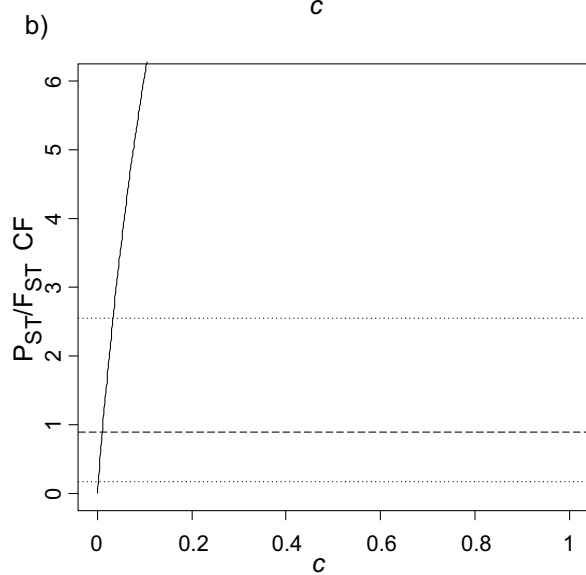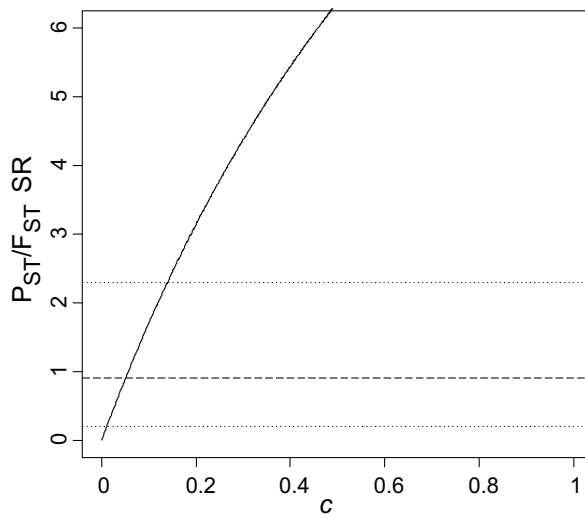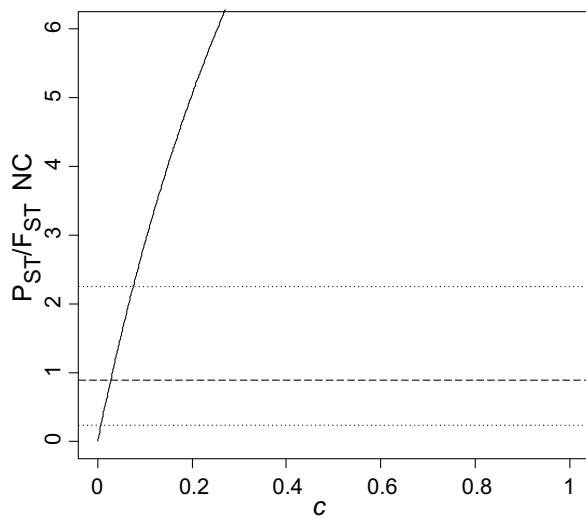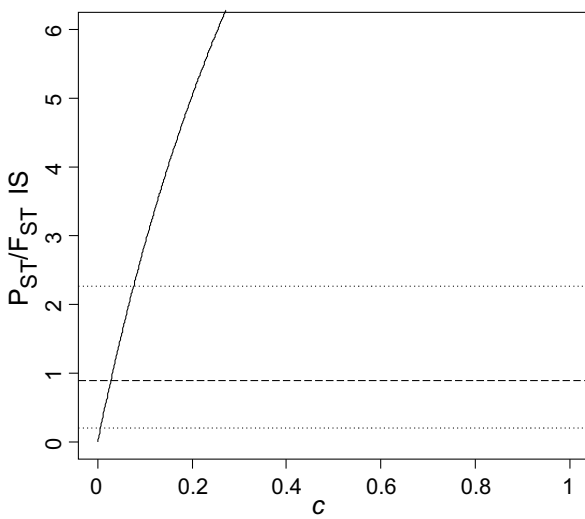

Supplement: Supplementary file 2 — Figure S2. Relationship between observed (solid line) and neutral (dashed line) PST/FST, for four phenotypic traits of (a) brown trout and (b) rainbow trout. [file eva0007-0921-sd2.pdf]
