# Supplementary material for: Contrasting patterns of genetic and phenotypic differentiation in two invasive salmonids in the southern hemisphere
Source: Evol Appl. 2014 Jul 23;7(8):921–36. doi: 10.1111/eva.12188 (PMC4211722; doi:10.1111/eva.12188)
Supplement: Supplementary file 3 — Table S1. Strains of rainbow trout and brown trout introduced in Chile and the Falkland Islands. [file eva0007-0921-sd3.docx]

**Table S1.** Strains of rainbow trout and brown trout introduced in Chile and the Falkland Islands (sources: MacCrimmon 1971; MacCrimmon and Marshall 1968; Arrowsmith and Pentelow 1965; Faundez et al. 1997; Colihueque et al. 2001; McDowall et al. 2001; Lhorente 2011).

| Country /Species | Origin | Strain/Population |
| --- | --- | --- |
|  |  |  |
| **Chile** |  |  |
| *Rainbow trout* | Germany | USA (probably steelhead from California) |
|  | Denmark | Cofradex |
|  |  | Hansen |
|  |  | Sangild |
|  |  | Jutland |
|  |  | Ollerupgård |
|  |  | Troutex Trachsel |
|  |  | AquaSearch Fresh |
|  |  | AquaSearch Late |
|  |  | AquaSearch Salt |
|  | USA | Troutlodge Kamloops |
|  |  | Troutlodge Silver Steelhead |
|  |  | Troutlodge McLeary |
|  |  | Donaldson |
|  | Norway | AquaGen/NLA |
|  |  | SalmoBreed |
|  | Finland | Arvo-kala |
|  |  | FGFRI/MTT |
|  |  |  |
| *Brown trout* | Germany | Hamburg (probably R. Elbe) |
|  | England | Unknown |
|  | USA | European stocks |
|  | Argentina | European stocks |
|  | Canada | European stocks |
|  |  |  |
| **Falkland Islands** |  |  |
| *Brown trout* | England | Surrey Trout Farm (Mr. Thomas Andrews, Guildford, R. Wey; Donald Leney) |
|  |  | Lancashire (probably R. Lune) |
|  | Scotland | Pentlands Hatchery |
|  | Chile | Lautaro hatchery (probably of German origin) |
|  | USA | Unknown (probably of German origin) |
|  |  |  |
