# Supplementary material for: Contrasting patterns of genetic and phenotypic differentiation in two invasive salmonids in the southern hemisphere
Source: Evol Appl. 2014 Jul 23;7(8):921–36. doi: 10.1111/eva.12188 (PMC4211722; doi:10.1111/eva.12188)
Supplement: Supplementary file 4 — Table S2. Summary genetic statistics for brown trout considering (a) global sample (over all populations) and (b) population-specific. [file eva0007-0921-sd4.docx]

**Table S2**. Summary genetic statistics for brown trout considering (a) global sample (over all populations) and (b) population-specific. All = all 14 markers; non-genic = 12 markers unlinked to genes; genic = 2 markers linked to genes (*MCHI* and *SasaTAP2A*).

(a)

| Parameter | All | Non-genic | Genic |
| --- | --- | --- | --- |
|  |  |  |  |
| *H*_O_ | 0.601 | 0.602 | 0.599 |
| *H*_E_ | 0.609 | 0.607 | 0.619 |
| Global *F_S_*_T_ | 0.191 | 0.195 | 0.165 |
| Chile *F*_ST_ | 0.087 | 0.083 | 0.110 |
| Falklands *F*_ST_ | 0.235 | 0.237 | 0.224 |
|  |  |  |  |
| Difference in diversity between Chile & Falklands | No | No | No |
| Differences in relatedness & *F*_ST_ between Chile & Falklands | No | No | No |
|  |  |  |  |

(b)

| River | *H*_o_ | | | *H*_e_ | | | *AR* | | | *N_A_* | | | *F*_IS_ | | |
| --- | --- | --- | --- | --- | --- | --- | --- | --- | --- | --- | --- | --- | --- | --- | --- |
|  | all | non-genic | genic | all | non-genic | genic | all | non-genic | genic | all | non-genic | genic | all | non-genic | Genic |
|  |  |  |  |  |  |  |  |  |  |  |  |  |  |  |  |
| GolGol | 0.66 | 0.66 | 0.69 | 0.66 | 0.67 | 0.64 | 4.67 | 4.95 | 4.27 | 4.8 | 5.75 | 4.50 | -0.005 | 0.008 | -0.087 |
| Butalcura | 0.61 | 0.60 | 0.70 | 0.63 | 0.62 | 0.71 | 4.11 | 4.31 | 3.50 | 4.19 | 4.83 | 3.50 | 0.027 | 0.029 | 0.018 |
| Bco-Enco | 0.66 | 0.67 | 0.66 | 0.65 | 0.65 | 0.69 | 4.35 | 4.44 | 5.14 | 4.46 | 5.00 | 5.50 | -0.015 | -0.027 | 0.055 |
| Pangal | 0.62 | 0.64 | 0.53 | 0.62 | 0.63 | 0.56 | 3.72 | 3.91 | 2.99 | 3.78 | 4.33 | 3.00 | -0.006 | -0.014 | 0.045 |
| Encanto | 0.62 | 0.63 | 0.55 | 0.65 | 0.67 | 0.53 | 4.44 | 4.72 | 3.83 | 4.56 | 5.42 | 4.00 | 0.050 | 0.061 | -0.031 |
| Bonito | 0.68 | 0.67 | 0.74 | 0.67 | 0.67 | 0.68 | 4.59 | 4.83 | 3.98 | 4.69 | 5.42 | 4.00 | -0.015 | -0.002 | -0.097 |
| Estancia | 0.72 | 0.75 | 0.54 | 0.76 | 0.77 | 0.73 | 6.21 | 6.62 | 5.89 | 6.44 | 8.17 | 6.50 | 0.055 | 0.023 | 0.260 |
| Finlay | 0.41 | 0.40 | 0.48 | 0.41 | 0.40 | 0.50 | 2.54 | 2.51 | 3.21 | 2.57 | 2.67 | 3.50 | 0.007 | -0.002 | 0.052 |
| Sarnys | 0.41 | 0.39 | 0.50 | 0.41 | 0.39 | 0.52 | 2.84 | 2.92 | 3.00 | 2.91 | 3.17 | 3.00 | -0.001 | -0.010 | 0.039 |
