# Supplementary material for: Contrasting patterns of genetic and phenotypic differentiation in two invasive salmonids in the southern hemisphere
Source: Evol Appl. 2014 Jul 23;7(8):921–36. doi: 10.1111/eva.12188 (PMC4211722; doi:10.1111/eva.12188)
Supplement: Supplementary file 5 — Table S3. Results of bayescan and lositan programs for outlier detection in (a) brown trout and (b) rainbow trout. [file eva0007-0921-sd5.docx]

**Table S3**. Results of BayeScan and LOSITAN programs for outlier detection in (a) brown trout and (b) rainbow trout.

1. brown trout

| Locus | BayeScan | | | | | LOSITAN | | | |
| --- | --- | --- | --- | --- | --- | --- | --- | --- | --- |
|  | *P* | log10(PO) | Alpha | *F*_ST_ | Selection | Het | *F*_ST_ | *P* | Selection |
|  |  |  |  |  |  |  |  |  |  |
| Str73 | 0.474 | -0.045 | -0.526 | 0.162 | *balancing* | 0.474 | 0.038 | 0.004 | *balancing* |
| Ssa408 | 0.020 | -1.690 | 0.001 | 0.217 | no | 0.909 | 0.193 | 0.688 | No |
| Ssa171 | 0.024 | -1.601 | -0.002 | 0.217 | no | 0.844 | 0.175 | 0.279 | No |
| SsoSL417 | 0.024 | -1.605 | -0.001 | 0.217 | no | 0.847 | 0.151 | 0.148 | No |
| Str15 | 0.034 | -1.448 | 0.003 | 0.218 | no | 0.741 | 0.311 | 0.748 | No |
| Ssa60 | 0.428 | -0.125 | 0.371 | 0.279 | no | 0.583 | 0.399 | 0.903 | No |
| Ssa410UoS | 0.312 | -0.342 | -0.156 | 0.197 | no | 0.921 | 0.227 | 0.894 | No |
| SsaF43 | 0.023 | -1.620 | 0.001 | 0.217 | no | 0.832 | 0.239 | 0.609 | No |
| BG935488 | 0.047 | -1.303 | -0.013 | 0.216 | no | 0.704 | 0.096 | 0.002 | *balancing* |
| SsaD71 | 0.024 | -1.598 | 0.005 | 0.218 | no | 0.823 | 0.186 | 0.350 | no |
| ppStr-3 | 0.225 | -0.536 | -0.166 | 0.198 | no | 0.663 | 0.157 | 0.098 | no |
| Ssa197 | 0.037 | -1.408 | 0.010 | 0.219 | no | 0.778 | 0.239 | 0.513 | no |
| SasaTAP2A | 0.033 | -1.466 | -0.001 | 0.217 | no | 0.768 | 0.191 | 0.221 | no |
| MHCI | 0.036 | -1.427 | 0.006 | 0.218 | no | 0.712 | 0.139 | 0.029 | no |

1. rainbow trout

| Locus | BayeScan | | | | | LOSITAN | | | |
| --- | --- | --- | --- | --- | --- | --- | --- | --- | --- |
|  | *P* | log10(PO) | Alpha | *F*_ST_ | Selection | Het | *F*_ST_ | *P* | Selection |
|  |  |  |  |  |  |  |  |  |  |
| OMM3089 | 0.991 | 2.052 | -1.250 | 0.073 | *balancing* | 0.683 | 0.065 | 0.328 | No |
| OMM1051 | 0.976 | 1.617 | -0.918 | 0.095 | *balancing* | 0.886 | 0.079 | 0.534 | No |
| OMM5188 | 0.891 | 0.911 | -0.734 | 0.111 | no | 0.820 | 0.084 | 0.649 | No |
| OMM5047 | 0.990 | 1.996 | -1.133 | 0.079 | *balancing* | 0.814 | 0.096 | 0.825 | No |
| OMM1741 | 0.992 | 2.093 | -1.1700 | 0.077 | *balancing* | 0.777 | 0.045 | 0.030 | No |
| OMM1008 | 0.981 | 1.713 | -1.037 | 0.086 | *balancing* | 0.769 | 0.091 | 0.717 | No |
| OMM1097 | 0.986 | 1.845 | -1.036 | 0.085 | *balancing* | 0.917 | 0.078 | 0.575 | No |
