# Supplementary material for: Contrasting patterns of genetic and phenotypic differentiation in two invasive salmonids in the southern hemisphere
Source: Evol Appl. 2014 Jul 23;7(8):921–36. doi: 10.1111/eva.12188 (PMC4211722; doi:10.1111/eva.12188)
Supplement: Supplementary file 6 — Table S4. (a) Differentiation of brown trout populations expressed as (a) pairwise FST comparisons (below diagonal) and Dest (above diagonal) among Chilean and Falklands populations and (b) as pairwise FST estimated from neutral (below diagonal) and gene-linked (above diagonal) markers. [file eva0007-0921-sd6.docx]

**Table S4.** (a) Differentiation of brown trout populations expressed as (a) pairwise *F_ST_* comparisons (below diagonal) and *D_est_* (above diagonal) among Chilean and Falklands populations and (b) as pairwise *F_ST_* estimated from neutral (below diagonal) and gene-linked (above diagonal) markers. Bold text indicates significance (*P*< 0.001) based on 10,000 permutations.

1. pairwise *F_ST_* and *D_est_* comparisons

|  | GolGol | Butalcura | Blo Enco | Pangal | Encanto | Bonito | Estancia | Finlay | Sarnys |
| --- | --- | --- | --- | --- | --- | --- | --- | --- | --- |
| GolGol | - | **0.083** | **0.105** | **0.153** | **0.093** | **0.070** | **0.224** | **0.484** | **0.490** |
| Butalcura | **0.054** | - | **0.111** | **0.162** | **0.099** | **0.056** | **0.237** | **0.404** | **0.392** |
| Blo Enco | **0.058** | 0.082 | - | **0.229** | **0.138** | **0.107** | **0.223** | **0.562** | **0.601** |
| Pangal | **0.117** | **0.121** | **0.133** | - | **0.223** | **0.120** | **0.210** | **0.556** | **0.483** |
| Encanto | **0.074** | **0.098** | **0.100** | 0.141 | - | **0.059** | **0.201** | **0.487** | **0.510** |
| Bonito | **0.045** | **0.057** | **0.071** | **0.081** | **0.044** | - | **0.205** | **0.530** | **0.566** |
| Estancia | **0.095** | **0.105** | **0.096** | **0.112** | **0.101** | **0.079** | - | **0.465** | **0.494** |
| Finlay | **0.355** | **0.341** | **0.390** | **0.387** | **0.354** | **0.350** | **0.270** | - | **0.021** |
| Sarnys | **0.340** | **0.318** | **0.384** | **0.352** | **0.347** | **0.340** | **0.261** | **0.068** | - |

1. *F_ST_* comparisons based on neutral (below diagonal) or gene-linked markers (above diagonal)

|  | GolGol | Butalcura | Blo Enco | Pangal | Encanto | Bonito | Estancia | Finlay | Sarnys |
| --- | --- | --- | --- | --- | --- | --- | --- | --- | --- |
| GolGol | - | **0.054** | **0.054** | **0.113** | **0.082** | **0.042** | **0.083** | **0.377** | **0.360** |
| Butalcura | **0.058** | - | **0.091** | **0.119** | **0.089** | **0.056** | **0.109** | **0.374** | **0.353** |
| Blo Enco | **0.082** | 0.027 | - | **0.119** | **0.093** | **0.075** | **0.095** | **0.424** | **0.412** |
| Pangal | **0.139** | **0.132** | **0.214** | - | **0.131** | **0.066** | **0.098** | **0.387** | **0.363** |
| Encanto | **0.016** | **0.148** | **0.145** | **0.208** | - | **0.038** | **0.083** | **0.361** | **0.350** |
| Bonito | **0.065** | **0.066** | **0.051** | **0.163** | **0.082** | - | **0.073** | **0.370** | **0.351** |
| Estancia | **0.1659** | **0.0812** | **0.0997** | **0.1944** | **0.2062** | **0.114** | - | **0.271** | **0.265** |
| Finlay | **0.2038** | **0.1037** | **0.1274** | **0.3891** | **0.3102** | **0.222** | **0.266** | - | **0.059** |
| Sarnys | **0.1938** | **0.0731** | **0.1768** | **0.2799** | **0.3308** | **0.272** | **0.240** | 0.103 | - |
