# Supplementary material for: Contrasting patterns of genetic and phenotypic differentiation in two invasive salmonids in the southern hemisphere
Source: Evol Appl. 2014 Jul 23;7(8):921–36. doi: 10.1111/eva.12188 (PMC4211722; doi:10.1111/eva.12188)
Supplement: Supplementary file 7 — Table S5. Correlations between PST, FST, PST/FST and geographical distance. [file eva0007-0921-sd7.docx]

**Table S5.** Correlations between *P*_ST_, *F*_ST_, *P*_ST_/*F*_ST_ and geographical distance.

| Species | Trait | Matrix 1 | Matrix 2 | Mantel *r* | *P* |
| --- | --- | --- | --- | --- | --- |
|  | |  |  |  |  |
|  | |  |  |  |  |
| Brown trout | CF | *P*_ST_ | *F*_ST_ | 0.714 | 0.201 |
|  |  | *P*_ST_ | Distance | -0.143 | 0.576 |
|  |  | *P*_ST_/*F*_ST_ | Distance | -0.142 | 0.591 |
|  |  |  |  |  |  |
|  | FWIS | *P*_ST_ | *F*_ST_ | 0.236 | 0.201 |
|  |  | *P*_ST_ | Distance | -0.309 | 0.871 |
|  |  | *P*_ST_/*F*_ST_ | Distance | -0.309 | 0.885 |
|  |  |  |  |  |  |
|  | FWSR | *P*_ST_ | *F*_ST_ | -0.333 | 0.935 |
|  |  | *P*_ST_ | Distance | -0.030 | 0.584 |
|  |  | *P*_ST_/*F*_ST_ | Distance | -0.039 | 0.504 |
|  |  |  |  |  |  |
|  | NCFW | *P*_ST_ | *F*_ST_ | -0.236 | 0.581 |
|  |  | *P*_ST_ | Distance | -0.006 | 0.462 |
|  |  | *P*_ST_/*F*_ST_ | Distance | -0.054 | 0.518 |
|  | |  |  |  |  |
| Rainbow trout | CF | *P*_ST_ | *F*_ST_ | -0.275 | 0.919 |
|  |  | *P*_ST_ | Distance | 0.417 | 0.069 |
|  |  | *P*_ST_/*F*_ST_ | Distance | -0.021 | 0.527 |
|  |  |  |  |  |  |
|  | FWIS | *P*_ST_ | F_ST_ | -0.038 | 0.556 |
|  |  | *P*_ST_ | Distance | -0.237 | 0.838 |
|  |  | *P*_ST_/*F*_ST_ | Distance | -0.239 | 0.857 |
|  |  |  |  |  |  |
|  | FWSR | *P*_ST_ | *F*_ST_ | 0.015 | 0.321 |
|  |  | *P*_ST_ | Distance | 0.252 | 0.139 |
|  |  | *P*_ST_/*F*_ST_ | Distance | 0.187 | 0.193 |
|  |  |  |  |  |  |
|  | NCFW | *P*_ST_ | *F*_ST_ | 0.025 | 0.476 |
|  |  | *P*_ST_ | Distance | 0.258 | 0.161 |
|  |  | *P*_ST_/*F*_ST_ | Distance | 0.180 | 0.252 |
|  |  |  |  |  |  |
